# Tissue specificity of oncogenic BRAF targeted to lung and thyroid through a shared lineage factor

**DOI:** 10.1016/j.isci.2023.107071

**Published:** 2023-06-08

**Authors:** Elin Schoultz, Shawn Liang, Therese Carlsson, Stefan Filges, Anders Ståhlberg, Henrik Fagman, Clotilde Wiel, Volkan Sayin, Mikael Nilsson

**Affiliations:** 1 Sahlgrenska Center for Cancer Research, University of Gothenburg, Göteborg, Sweden; 2 Department of Medical Chemistry and Cell Biology, Institute of Biomedicine, University of Gothenburg, Göteborg, Sweden; 3 Department of Laboratory Medicine, Institute of Biomedicine, University of Gothenburg, Göteborg, Sweden; 4 Department of Surgery, Institute of Clinical Sciences, University of Gothenburg, Göteborg, Sweden; 5 Region Västra Götaland, Sahlgrenska University Hospital, Department of Clinical Genetics and Genomics, Göteborg, Sweden; 6 Wallenberg Centre for Molecular and Translational Medicine, University of Gothenburg, Göteborg, Sweden

**Keywords:** Genetics, Cell biology, Cancer

## Abstract

Cells of origin in cancer determine tumor phenotypes, but whether lineage-defining transcription factors might influence tissue specificity of tumorigenesis among organs with similar developmental traits are unknown. We demonstrate here that tumor development and progression markedly differ in lung and thyroid targeted by *Braf* mutation in *Nkx2.1CreER*^*T2*^ mice heterozygous for *Nkx2-1*. In absence of tamoxifen, non-induced *Nkx2.1CreER*^*T2*^*;Braf*^*CA/+*^ mutants developed multiple full-blown lung adenocarcinomas with a latency of 1–3 months whereas thyroid tumors were rare and constrained, although minute *Braf*^*CA*^ activation documented by variant allele sequencing was similar in both tissues. Induced oncogene activation accelerated neoplastic growth only in the lungs. By contrast, NKX2-1^+^ progenitor cells were equally responsive to constitutive expression of mutant Braf during lung and thyroid development. Both lung and thyroid cells transiently downregulated NKX2-1 in early tumor stages. These results indicate that BRAF^V600E^-induced tumorigenesis obey organ-specific traits that might be differentially modified by a shared lineage factor.

## Highlights

• Tissue-specific response to BRAF^V600E^ conditionally targeted by the *Nkx2-1* promoter
• Tumorigenesis depends on equally low incidence of spontaneous oncogene activation
• Multiple lung tumors rapidly progress to invasive adenocarcinomas of alveolar origin
• Thyroid tumors are extremely rare and confined to indolent papillary microcarcinomas

## Graphical abstract

**Figure:**
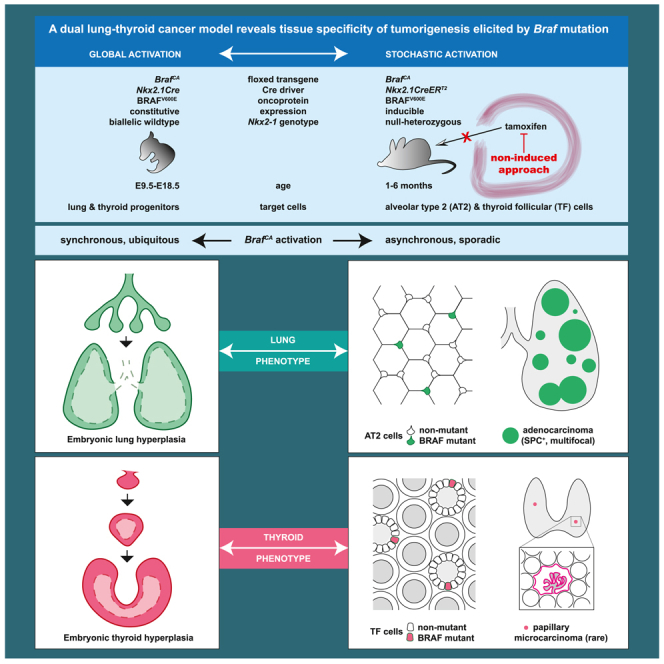

## Introduction

Although targeted oncogene activation has been invaluable to understand organ-specific carcinogenesis there are several methodological constraints that limit the usefulness of transgenic mouse models in reproducing sporadic tumorigenesis in humans. First, as a means of conditional targeting the *Cre* driver is usually expressed in differentiated cells, which might not be representative for the cell-of-origin of cancer. Moreover, both constitutive and induced oncogene expression involve the majority of targeted cells already from the start of tumor initiation, which impairs tracing of clonal tumor development and progression in a natural tissue environment. For example, viral vector-based administration of a Cre-activated mutant *Braf* allele (*Braf*^*CA*^) into the bronchial tree results in widespread development of myriads of BRAF^V600E^-induced lung tumors on the expense of normal lung parenchyma and the animals die of respiratory distress before progression to carcinoma.^1^^,^^2^ In the thyroid, conditional activation of mutant BRAF readily triggers tumor formation but at the same time the follicular epithelial cells globally dedifferentiate and stop producing thyroid hormone, which in fact makes it difficult to distinguish neoplastic from goitrogenic growth resulting from hypothyroidism.^3^

We recently characterized a BRAF^V600E^-driven mouse thyroid cancer model that displayed a limited number of tumor initiation *loci* and allowed monitoring of tumor development and progression with maintained systemic thyroid function until high age.^4^ Key to modeling sporadic tumorigenesis in the thyroid was a low but significant basal rate of spontaneous recombination – as commonly referred to “Cre leakiness” – by an inducible Cre driver linked to the thyroglobulin promoter (*Tg-CreER*^*T2*^), which in the absence of inducing agent (tamoxifen) activated *Braf*^*CA*^ in a minority of targeted cells. Notably, this study also showed that immature thyroid cells in the developing gland possessed much higher tumorigenic potential than adult BRAF mutant follicular epithelial cells, suggesting that tissue maturity profoundly influenced tumor cell behavior and ultimately mutation penetrance. Stochastic *Braf*^*CA*^ activation in but a few cells at a time prevailing in non-induced conditions thus implies a general rationale to overcome bias because of ubiquitous BRAF^V600E^ expression, and provides for a more reliable investigation of tumor clonality and heterogeneity generated in a preserved tissue microenvironment.

*Nkx2-1* is a developmental gene required for normal morphogenesis in both thyroid and lung in mice.^5^ A dual dependence of this transcription factor in organ development is underscored by the coincidence of congenital hypothyroidism and neonatal respiratory distress in humans with monoallelic loss of *NKX2-1*.^6^ Onset of NKX2-1 expression occurs simultaneously in thyroid and lung lineage progenitors already from the start of organogenesis in foregut endoderm.^7^ However, whereas Nkx2-1 continuously promotes embryonic thyroid growth and differentiation involving essentially all lineage progeny, in the developing lung Nkx2-1 transcriptional activity is largely confined to the distal branching airways and eventually in alveolar differentiation.^8^ Targeted oncogene activation using *Nkx2-1* as Cre driver might thus potentially uncover organotypic features of tumor development related to spatiotemporally different expression patterns in thyroid and lung, respectively, which come into place embryonically and maintain in adulthood. It is noteworthy that whereas *NKX2-1* is an established lung cancer gene with pleiotropic actions,^9^ it is unknown to what extent, if any, Nkx2-1 might influence carcinogenesis and tumor progression in the thyroid gland.

In humans, *BRAF(V600E)* is by far the most common driver mutation in thyroid cancer mainly comprising papillary thyroid carcinoma (PTC),^10^^,^^11^ whereas less than 5% of non-small cell lung carcinoma (NSCLC) carry this mutation.^10^^,^^11^ On the other hand, most BRAF-induced PTCs progress slowly and may escape detection and diagnosis for decades, whereas in general BRAF^+^ lung tumors are highly malignant. In what extent such large organ differences associated with the same oncogenic mutation depend on tissue-specific factors that might influence mutation penetrance is largely unknown. Here, we addressed this issue by monitoring and comparing tumor prevalence and tumor features correlated with the actual number of cells carrying an activated *Braf* mutant allele, primarily targeted to thyroid and lung by the *Nkx2-1* promoter, in *Nkx2.1-CreER*^*T2*^*;Braf*^*CA/+*^ mice. Results unequivocally showed striking organ differences in favor of lung carcinogenesis although the incidence of spontaneous *Braf*^*CA*^ activation in non-induced conditions, estimated by ultrasensitive sequencing, was similar for thyroid and lung tissues. Because the *Nkx2.1-CreER*^*T2*^ driver was designed to abolish *Nkx2-1* gene function^12^ we hypothesize that *Nkx2-1* gene dosage might differentially modify tumor development and progression in a lineage-dependent manner.

## Results

### BRAF^V600E^ targeted to Nkx2-1-lineage cells confers embryonic thyroid and lung hyperplasia and perinatal lethality

**Figure 1:**
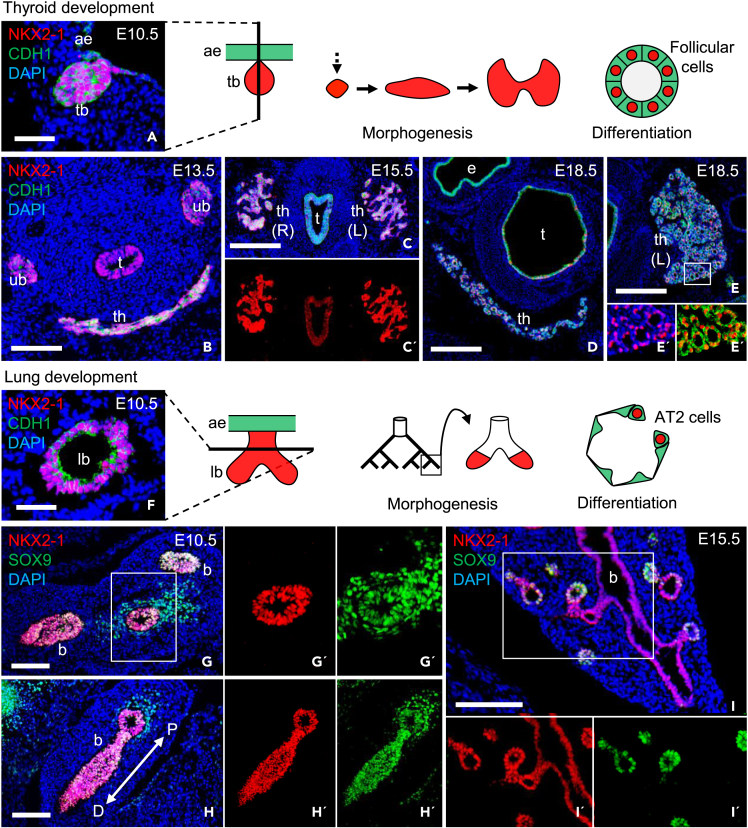
Nkx2-1 expression in the developing thyroid and lung in mice Immunostaining of NKX2-1 (red) and CDH1 or SOX9 (green). (A) Thyroid bud (left) and schematic representation of developmental stages (right). (B–E) Embryonic thyroid: midline primordium after descent (B), lobulation stage (C, C′) and, orthotopically, the isthmus (D) and lateral lobe (E, E′). (F) Lung bud (left) and schematic representation of developmental stages (right). (G–I) Lung branching morphogenesis: early (G-H′) and late (I-I′) stages. Monochromic images C′, G′ (boxed area in G), H′ (boxed area in H) and I′ provided for clarity. DAPI staining visualized cell nuclei. th, thyroid; ub, ultimobranchial body; t, trachea, b, bronchus; e, esophagus; L, left; R, right; P, proximal; D, distal; E, embryonic day. Scale bars: 500 (D, E), 200 (C), 100 (B, G-I) and 50 (A, F) μm.

**Figure 2:**
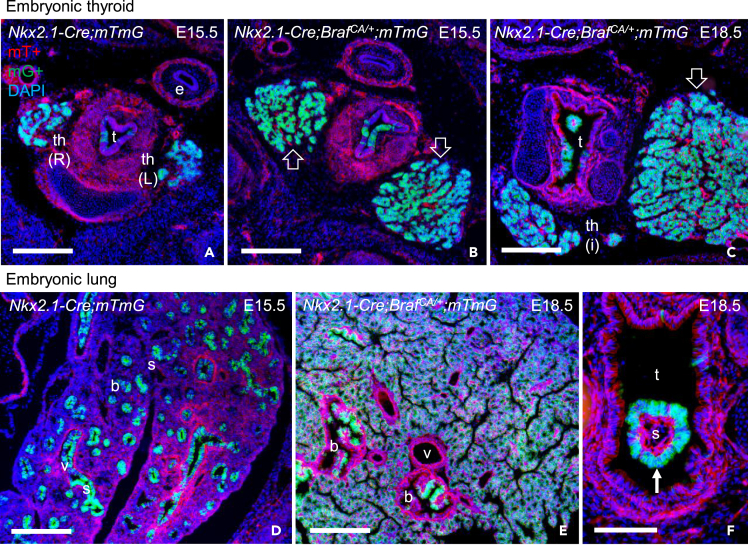
Clonal tracing of BRAF mutant *Nkx2-1*-lineage cells in embryonic thyroid and lung Images based on constitutive activation of the double-fluorescent *mTmG* reporter gene in *Nkx2.1-Cre;mTmG* (control) and *Nkx2.1-Cre;Braf*^*CA/+*^*;mTmG* (mutant) mice. Cells are default-labeled with mTomato (mT^+^) and GFP-labeled (mG^+^) on activation. (A and B) Normal (A) and Braf-mutant (B) thyroid at E15.5. (C) Braf-mutant thyroid at 18.5. Note enlarged lobes in mutants (open arrows). (D) Normal lung at E15.5. (E and F) Braf-mutant lung (E) and proximal airway (F) at E18.5. Note ubiquitous mG-labeling of hyperplastic alveolar tissue and polypoid tumors in bronchial airways and trachea (arrow). th, thyroid; I, isthmus; L, left lobe; R, right lobe; t, trachea; e, esophagus; b, bronchus; s, stroma; v, vessel. Scale bars: 200 (A-E) and 100 (F) μm. See also Figure S1.

NKX2-1 is ubiquitously expressed during thyroid development^13^ (Figures 1A–1E′) and in lung branching morphogenesis (Figures 1F–1I′), consistent with essentially all parenchymal cells are potentially targeted by Cre-mediated recombination using the *Nkx2-1* promoter as Cre driver. On this basis, we generated *Nkx2.1-Cre;Braf*^*CA/+*^*;mTmG* mouse embryos (by crossing previously described strains:^1^^,^^2^) to investigate the response of thyroid and lung progenitor cells to constitutive *Braf*^*CA*^ activation by clonal tracing. Concomitant reporter gene activation readily showed that conditional knock-in of the mutant *Braf* allele resulted in progressive hyperplastic growth of the thyroid lobes (Figures 2A–2C). Volumetry indicated that thyroid size increased four times in E18.5 mutants as compared to wildtype littermates (n = 3 for both genotypes). Similarly, mutant lungs displayed massive hyperplasia that impaired embryonic development of alveoli (Figures 2D and 2E). On the other hand, neoplastic growth in the proximal airways was restricted to focal polyps whereas the remaining mucosa appeared normal and did not recombine the reporter gene (Figures 2C and 2F). Mosaic reporter activation was also evident in the developing lung but not in the thyroid of normal mice (Figures S1A and S1B). Altogether, this suggested that conditional *Braf*^*CA*^ activation was less efficient in lung progenitors, possibly related to Nkx2-1 expression in favor of distal cells as branching progressed. No other cell types – in thyroid, lung, or other nearby embryonic tissues – were trace-labeled or showed aberrant growth indicating specificity of oncogene targeting to *Nkx2-1*-lineage cells only. *Nkx2.1-Cre;Braf*^*CA/+*^ mice did not survive after birth because of the severe lung phenotype.

### Distinct tumorigenic effects of an inducible *Braf*^*CA*^ allele in thyroid and lung tissues with similar rates of spontaneous Cre-mediated activation in the absence of tamoxifen

Employing a thyroid-specific inducible *Cre* construct linked to the thyroglobulin (*Tg*) promoter,^14^ we previously found that by omitting tamoxifen, i.e., without induction, the spontaneous rate of Cre-mediated recombination is not negligible and that multifocal PTC tumors readily developed in non-induced *Tg-CreER*^*T2*^*;Braf*^*CA/+*^ mice.^4^ With this information at hand, we conducted a series of experiments to elucidate whether a *Nkx2.1-CreER*^*T2*^ driver originally developed for conditional targeting of Nkx2-1^+^ GABAergic neurons^12^ might possess Cre leakiness sufficient to trigger stochastic oncogene activation for comparison of sporadic tumorigenesis in the thyroid and lungs of *Nkx2.1-CreER*^*T2*^*;Braf*^*CA/+*^ mice.

**Figure 3:**
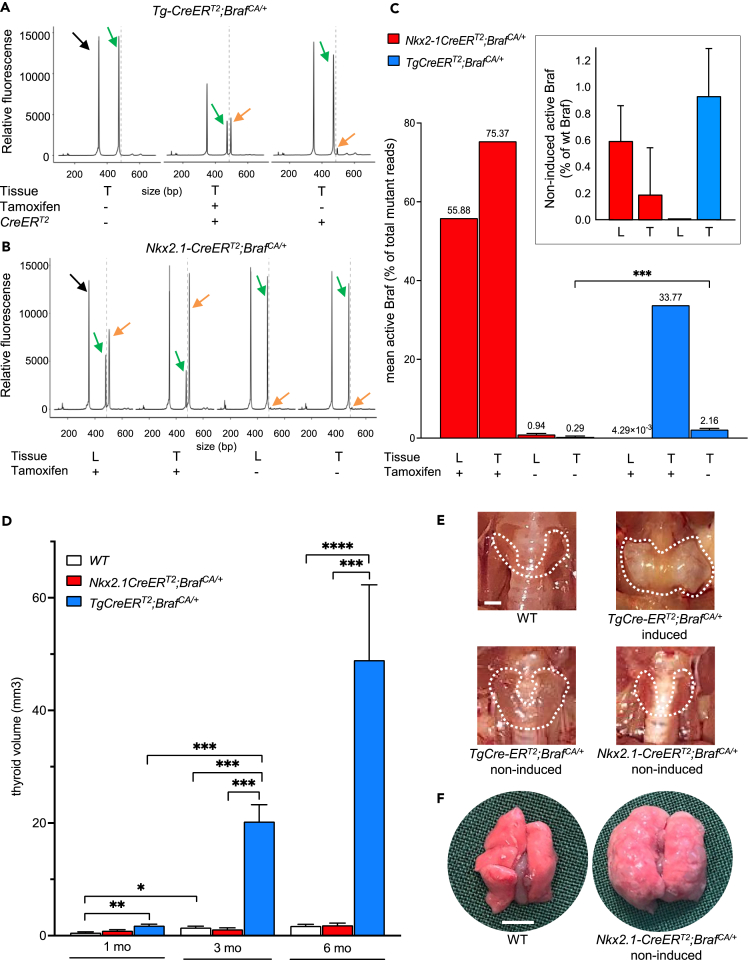
Tumorigenic response correlated with magnitude of spontaneous Cre-mediated *Braf*^*CA*^ recombination in adult thyroid and lung tissues Variant alleles (inactive and active) of mutant *Braf* were quantified by SiMSen-Seq analysis^15^ in *Nkx2.1-CreER*^*T2*^*;Braf*^*CA/+*^ and *Tg-CreER*^*T2*^*;Braf*^*CA/+*^ mice 10 days after induction with tamoxifen (x4 after weaning) or in age-matched littermates kept in non-induced conditions since birth. (A and B) Library preparation curves of wild type (wt) *Braf* (185 bp; black arrows), native *Braf*^*CA*^ (308 bp; green arrows) and activated *Braf*^*CA*^ (335 pp; orange arrows). (C) Prevalence of *Braf*^*CA*^ activation. Calculated data using Prism 9 by dividing activated *Braf*^*CA*^ reads with total (native + activated) *Braf*^*CA*^ reads. Mean ± SEM of: *Braf*^*CA/+*^ (n = 3); *Tg-CreER*^*T2*^*;Braf*^*CA/+*^, induced (n = 2), non-induced (n = 9); *Nkx2.1-CreER*^*T2*^*;Braf*^*CA/+*^, induced (n = 2), non-induced (n = 6); unpaired t-test (p value): ∗∗∗<0.0002. Inset shows non-induced data correlated with wt *Braf* level in the same samples. (D) Thyroid size changes in 1–6-month-old non-induced Braf mutant mice. Mean ± SEM (n/timepoint): WT (n = 6–8); *Nkx2.1-CreER*^*T2*^*;Braf*^*CA/+*^ (n = 6–8); *Tg-CreER*^*T2*^*;Braf*^*CA/+*^ (n = 8–10); one-way ANOVA tests (p values): ∗<0.0332, ∗∗<0.0.0021, ∗∗∗<0.0002 and ∗∗∗∗<0.0001. (E and F) Pathoanatomical changes in thyroid (encircled in E) and lungs (F) of 6-month-old mutants (genotypes indicated). Scale bars: 1 (E) and 5 (F) mm. L, lung; T, thyroid; *Tg*, thyroglobulin promoter. See also Table S1 and Figure S2.

First, to more precisely estimate the prevalence of Braf mutant thyroid and lung cells in non-induced versus induced conditions we employed a simplified assay for ultrasensitive mutation analysis (SiMSen-Seq^15^). The sequencing protocol was designed based on previous notions that *Braf*^*CA*^ obtains a unique allele sequence on recombination.^1^ Induction with tamoxifen was carried out immediately after weaning and tissues were sampled from both induced and non-induced mutants 10 days after the first injection. Sequencing readily identified wildtype *Braf* (185 bp), native *Braf*^*CA*^ (308 bp) and activated (tamoxifen-induced) *Braf*^*CA*^ (335 bp) alleles in both *Tg-CreER*^*T2*^*;Braf*^*CA/+*^ and *Nkx2.1-CreER*^*T2*^*;Braf*^*CA/+*^ mice (Figures 3A and 3B; left, middle and right panels). Induced activation by *Nkx2.1-CreER*^*T2*^ showed higher recombination rates in mutant thyroids (Figure 3C), possibly reflecting a lower level of mosaicism than in lung tissue as documented by lineage tracing (Figures S1A and S1B).

In absence of tamoxifen, both thyroid and lungs of *Nkx2.1-CreER*^*T2*^*;Braf*^*CA/+*^ mice showed measurable amounts of activated *Braf*^*CA*^ which however were smaller than the activated allele fraction found in the thyroid of non-induced *Tg-CreER*^*T2*^*;Braf*^*CA/+*^ mutants (Figures 3A–3C). Because the number of matching and total reads varied much depending on sample size (e.g. for non-induced mutants: 5000–15000 for total reads and 0–67 for active reads per sample), we correlated specific barcode families of mutant *Braf* to that of wildtype *Braf* to estimate the actual number of cells carrying the activated allele. This indicated that less than 1% of cells in both lung and thyroid expressed BRAF^V600E^ oncoprotein because of spontaneous Cre-mediated activation in 6-week-old mice irrespective of Cre driver (Figure 3C, inset). Overall, differences in mutant cell numbers were statistically significant only for thyroid tissue in *Nkx2.1-CreER*^*T2*^*;Braf*^*CA/+*^ and *Tg-CreER*^*T2*^*;Braf*^*CA/+*^ mice (Figure 3C). As expected, there were no signs of oncogene activation in the lungs using thyroglobulin as Cre driver.

Volumetric measurements of the thyroid *in situ* showed progressively increased lobe size – because of a combination of follicle enlargement and neoplastic growth^4^ – between 1 and 6 months in *Tg-CreER*^*T2*^*;Braf*^*CA/+*^ mice whereas, remarkably, thyroid size did not change compared to age-matched wildtype in *Nkx2.1-CreER*^*T2*^*;Braf*^*CA/+*^ mutants (Figures 3D and 3E). By contrast, the lungs in the latter mouse model were heavily congested, in comparison to the soft texture of wildtype lungs, and spotted with conspicuous subpleural tumors (Figure 3F).

Altogether, these observations indicated that equally sparse activation of mutant BRAF manufactured by leaky Cre activity generated striking tissue differences in tumor development. Next, we attempted to further characterize tumorigenesis in the dual lung-thyroid cancer model comparing non-induced and induced states.

### Asynchronous lung tumor development because of stochastic BRAF activation in *Nkx2.1-CreER*^*T2*^*;Braf*^*CA/+*^ mice

**Figure 4:**
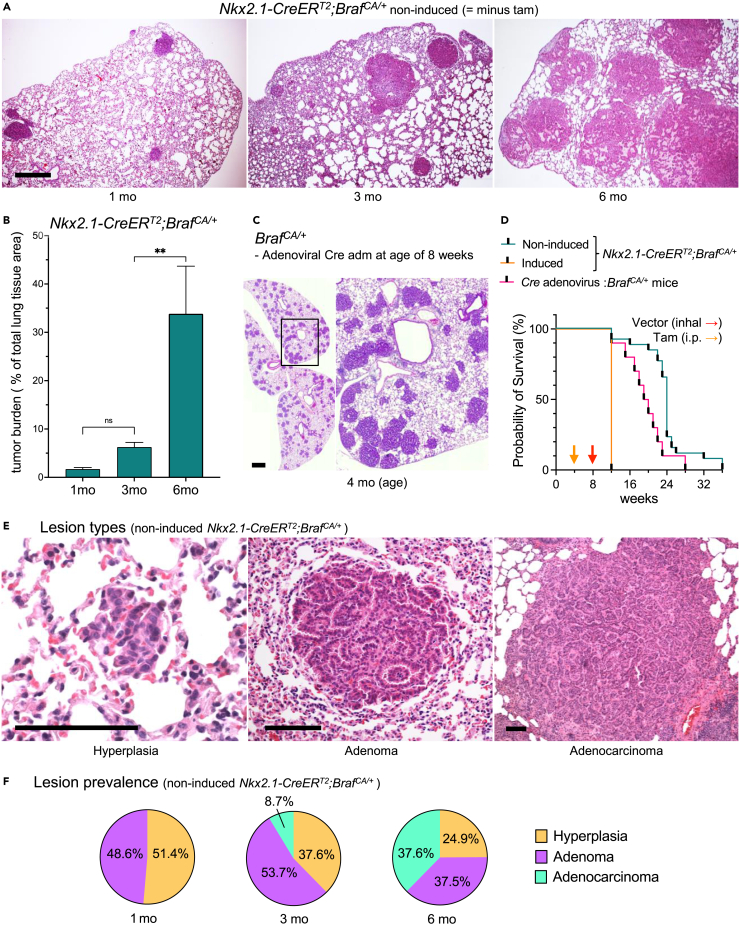
Sporadic lung tumor development and progression caused by stochastic *Braf*^*CA*^ activation in Nkx2-1-lineage cells All data obtained from non-induced *Nkx2.1-CreER*^*T2*^*;Braf*^*CA/+*^ mice except comparison to induced mice in C and D. (A) Multifocal tumor growth; overview of tumor size and shape at ages 1, 3, and 6 months (left, middle and right, representative images). (B) Tumor/lung tissue ratio; mean ± SD (n = 6 mice per time point; QuPath-based calculations from 3 cross-sections per lobe): unpaired t-test (p value): ∗∗<0.0021. (C) Synchronous lung adenoma development in *Braf*^*CA/+*^ mice after inhalation of Cre adenovirus; high resolution of boxed area to the right. (D) Kaplan-Meyer survival curves; asphyxia cause of death in all animals: *Nkx2.1-CreER*^*T2*^*;Braf*^*CA/+*^, induced (n = 5), non-induced (n = 25); *Braf*^*CA/+*^ (n = 15). Colored arrows indicate onset of induction with tamoxifen (Inj) and adenoviral Cre (Inhal), respectively. (E) Histopathology of lung tumors (left, middle and right). (F) Tumor staging scores; counts based on evaluation of random tissue sections (n = 18 per timepoint) also used in B. Scale bars: 1000 (C) and 100 (A and E) μm. See also Figures S3 and S4.

Evaluation of serial paraffin sections confirmed progressive lung tumor development with 100% penetrance (n = 30) in non-induced *Nkx2.1-CreER*^*T2*^*;Braf*^*CA/+*^ mice (Figures 4A and 4B). Neoplastic growth was evident at age 1 month (Figure 4A, left) but not at earlier time points (data not shown; based on sectioned tissues from E18.5, P0 and P10 mutants). SiMSen-Seq analysis confirmed lack of *Braf*^*CA*^ recombination in neonates (Figure S2), and only few reads (<5) of the active Braf mutant allele were encountered in some P10 lung samples (data not shown), collectively indicating that tumor initiation started postnatally.

Individual lung tumors varied much in diameter most notably at 3 months (Figure 4A, middle). Co-existing large tumors often coalesced and collectively occupied one-third or more of the entire lung lobe in 6-month-old mutants (Figure 4A, right). The largest tumor diameter was approximately 3 mm, although small tumors were also present in end-stage lungs.

The heterogeneous tumor phenotype markedly differed to that observed following disseminated activation of the mutant *Braf* allele through inhalation of an adenoviral *Cre* vector, which showed synchronous development of multiple small adenomas (Figure 4C). Moreover, comparison of survival rates revealed significant differences depending on means of oncogene activation with a median animal survival of 19 and 24 weeks in Cre-adenoviral versus non-induced conditions (Figure 4D and Table S1A). For comparison, mutants survived no longer than 8 weeks after induction with tamoxifen (Figure 4D). Respiratory distress because of pulmonary tumor burden was the reason for euthanasia in all groups.

These observations indicated that non-induced activation of mutant BRAF occurred stochastically in lung tissue leading to the formation of multifocal tumors that conceivably recapitulated the time course of sporadic lung carcinogenesis.

### Sporadic BRAF^V600E^-driven lung adenomas frequently progress to adenocarcinomas

The fact that survival varied from 12 to 36 weeks in non-induced *Nkx2.1-CreER*^*T2*^*;Braf*^*CA/+*^mice (Figure 4D and Table S1B) suggested that asynchronous tumor development conferred a wide spectrum of phenotypes and outcomes that might relate to different onset of growth and progression of individual tumors. Accordingly, lung lesions were histopathological heterogeneous comprising hyperplasia (Figure 4E, left), adenomas (Figure 4E, middle) and adenocarcinomas (Figure 4E, right) with altered occurrence over time consistent with a gradual progression toward a malignant phenotype (Figure 4F). Thus, benign lung tumors distinguished by small size, rounded shape and demarcated outer border constituted the majority of lesions at 1 and 3 months whereas adenocarcinomas defined by atypic cell morphology and invasive growth pattern were less frequent at age 3 months and numerous at age 6 months. The interior of advanced tumors regularly showed a papillary growth pattern with presence of a proteinaceous exudate in the luminal compartment (Figures S3A and S3B), whereas invasive tumor portions consisted of more solid extensions that infiltrated the surrounding alveolar tissue (Figures S3C–S3E). Both adenomas and carcinomas were mostly not associated with the bronchial tree. However, occasionally the bronchial epithelium was hyperplastic (Figure S4A), and some tumors also adhered to a bronchial wall although without any apparent transition site (Figure S4B). Advanced tumor stages also involved invasion into the bronchial lumen (Figures S4C and S4C′).

### Alveolar type 2 cell origin predominates in BRAF^V600E^-induced lung carcinogenesis

**Figure 5:**
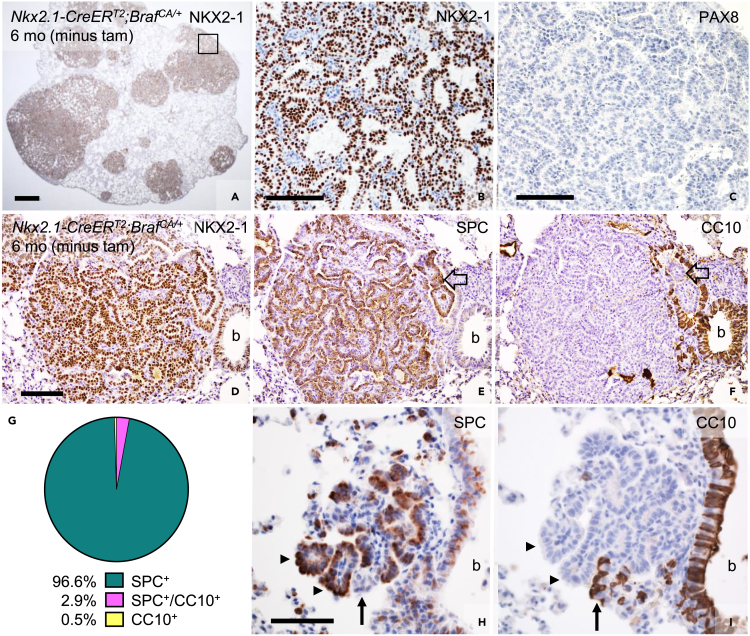
NKX2-1 and SPC co-expression in sporadically developed lung tumors Immunohistochemical staining of NKX2-1, PAX8, surfactant protein C (SPC) and club cell protein 10 (CC10) in lung tissue of non-induced *Nkx2.1-CreER*^*T2*^*;Braf*^*CA/+*^ mutants. (A–C) NKX2-1^+^/PAX8^-^ adenocarcinoma, overview (A) and closeup (B; boxed area in A and parallel section to C). (D–F) NKX2-1^+^/SPC^+^/CC10^-^ adenocarcinoma; from parallel sections. Open arrows indicate intrabronchial tumor portion. (G) Distribution of SPC^+^ only, CC10^+^ only and compound (SPC^+^/CC10^+^) lung tumors (n = 414, collectively from 3 section levels/lobe in 3 mutant mice). (H and I) Mutually exclusive expression of SPC and CC10 in a compound adenoma; from parallel sections. Arrow and arrowheads indicate cells stained for either marker. b, bronchiole. Scale bars: 500 (A), 100 (B-D) and 50 (H, I) μm. See also Figures S5–S8.

Lung adenocarcinoma cells were in general strongly positive for NKX2-1 (Figures 5A and 5B) but negative for the thyroid-specific transcription factor PAX8 (Figure 5C), which is consistent with histopathological features of NSCLC.^16^^,^^17^ There are several cell types that potentially may give rise to NSCLC and it is also evident that the differentiation state of mutated cells can influence tumor growth and the overall tumor phenotype.^18^ Moreover, because all lung epithelial cell types derive from a common embryonic NKX2-1^+^ ancestor pool, any lineage descendants could potentially be targeted with the *Nkx2.1-CreER*^*T2*^ driver. To get an idea whether miscellaneous cell targeting might explain tumor heterogeneity in the current lung cancer model we conduced immunohistochemical staining for club cell protein 10 (CC10) and surfactant protein C (SPC) predicted to label – based on the natural expression pattern (Figures S5A–S5C) – bronchiole- and alveolar type 2 (AT2)-derived BRAF mutant cells, respectively. This showed that the vast majority of lung tumors uniformly consisted of cells that co-expressed NKX2-1 and SPC and were negative for CC10 (Figures 5D–5G and S6A and S6B). Less than 3% were mixed tumors with mostly a minor part being CC10 positive (Figures 5G–5I), and very few, small tumors were predominated by CC10^+^ cells (Figures S6C and S6D). In all instances, SPC and CC10 expression were mutually exclusive with the exception of occasional CC10 negative tumors that showed only weak and heterogeneous SPC staining, i.e., many cells were negative for both markers (Figures S7A and S7B). Observations of a much more widespread proliferative response comprising both CC10^+^ bronchiolar and SPC^+^ alveolar cells already 10 days after induced activation with tamoxifen (Figures S8A–S8C) further argued that lung cancer development in non-induced *Nkx2.1-CreER*^*T2*^*;Braf*^*CA/+*^ mice predominantly derived from AT2 cells.

Notably, although NKX2-1 was overall strongly expressed in advanced lung tumor stages, small tumor *foci* presumably representing early lesions often showed a heterogeneous and overall reduced nuclear NKX2-1 immunoreactivity (Figure S7C). It was also evident that both NKX2-1 and SPC were lost in invasive carcinoma cells (Figures S7D, S7D′, S7E, and S7E′). In view of previous notions that SPC is transcriptionally regulated by Nkx2-1,^19^ these findings suggested that both *Nkx2-1* alleles are required for stable expression and, moreover, that constitutive BRAF signaling elicits downregulation of NKX2-1 accompanied by loss of SPC in certain lung tumor stages of *Nkx2.1-CreER*^*T2*^*;Braf*^*CA/+*^ mice.

### Sporadically developed lung tumors are monoclonal

**Figure 6:**
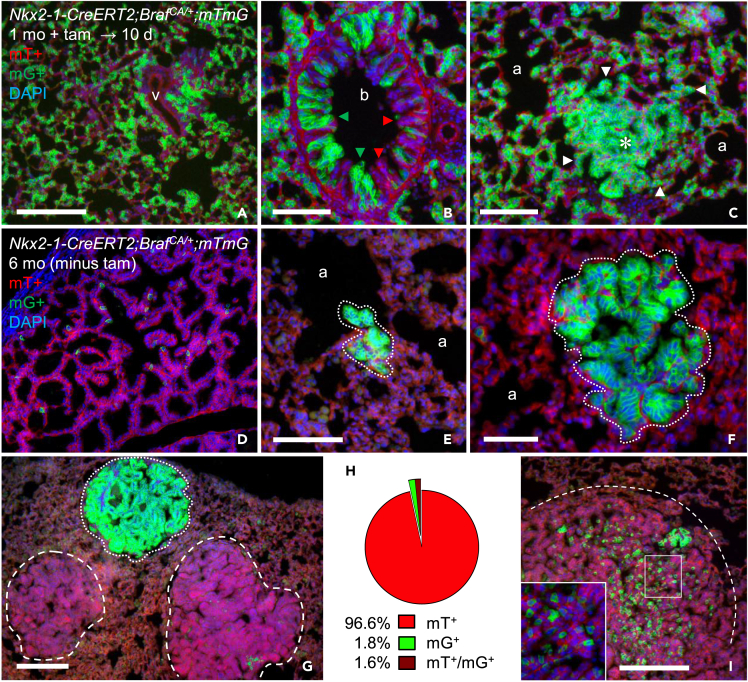
Clonal tracing of BRAF mutant lung cells *Nkx2.1-CreER*^*T2*^*;Braf*^*CA/+*^*;mTmG* mice were analyzed for *mTmG* reporter activation in induced (A-C; investigated 10 days (d) after tamoxifen (tam) injections x4) and non-induced (D-I) conditions. (A) General GFP-labeling and hyperplasia of alveolar tissue. (B) Mosaic GFP-labeling of hyperplastic bronchial epithelium; red and green arrowheads indicate mT^+^ and mG^+^ clones. (C) GFP-labeling of preformed adenoma; arrowheads indicate coalescence of adjacent G^+^ alveolar cells with tumor border. (D) Density of single mG^+^ alveolar cells undergoing spontaneous Cre-mediated reporter activation during the lifetime of 6 months (mo); lack of clonal expansion suggests these cells are non-mutant. (E–G) Homogeneous GFP-labeling of lung hyperplasia (E), adenoma (F), and adenocarcinoma (G) indicating monoclonal tumor growth (encircled). Note (in G) co-existing large tumors essentially devoid of mG^+^ cells. (H) Distribution of non-induced lung tumors with and without *Braf*^*CA*^ and *mTmG* co-activation (n = 437; collectively from 1-, 3- and 6-month-old mutants (n = 3 per age) and three section levels evaluated per lobe). (I) Heterogeneous GFP-labeling of a large lung tumor (partly outlined); inset shows high power of boxed area. Scattered mG^+^ cells presumably representing intratumor spread of a GFP-labeled tumor subclone are distinguished. a, alveolus; b, bronchiole; v, vessel. Scale bars: 200 (G, I) 100 (A-E), and 50 (F) μm.

To further characterize the sporadic lung cancer model, tumor clonality was investigated in *Nkx2.1-CreER*^*T2*^*;Braf*^*CA/+*^;*mTmG* mice with and without tamoxifen treatment. In 1-month-old mutants, induced co-activation of the *mTmG* reporter gene not only GFP-labeled most (if not all) AT2 cells but caused widespread alveolar hyperplasia already 10 days after induction (Figure 6A).

Bronchiolar labeling was heterogeneous in that many epithelial cells retained mTomato expression (Figure 6B), consistent with mosaic responsiveness to conditional reporter activation similar to the embryonic lung (Figure S1B′). Notably, preformed adenoma rudiments, which conspicuously coalesced with induced-proliferating AT2 cells, were uniformly GFP-labeled (Figure 6C). This indicated that Cre recombinase remained susceptible to tamoxifen after spontaneous *Braf*^*CA*^ recombination and expression of the oncogene in lung tissue.

In non-induced conditions, the low recombination rate predicts that co-activation of the reporter gene likely comprises a minority of targeted cells slowly accumulating over time. Indeed, mG^+^ alveolar cells were few and appeared mostly solitary without signs of clonal expansion (Figure 6D), suggesting *mTmG* activation predominantly occurred in non-mutant cells that did not proliferate. However, uniform GFP labeling of cells was occasionally evident in small clusters of neoplastic cells (Figure 6E), circumscribed adenomas (Figure 6F) and advanced-stage adenocarcinomas (Figure 6G). Such homogeneous mG^+^ labeling is consistent with monoclonal tumor development from an ancestral mutant cell in which *mTmG* was activated before or, perhaps less likely, concomitant with *Braf*^*CA*^ activation. By contrast, as previously shown,^4^ an oligoclonal origin would predict dual labeling of the tumor with complete separation of mG^+^ and mT^+^ clones evident early after tumor initiation. Lung tumors lacking signs of reporter activation and thus escaping clonal tracing were nonetheless frequent (Figures 6G and 6H). This does not necessarily mean that the mutant *Braf* allele is more easily activated than the reporter gene, because the likelihood of spontaneous co-activation is much smaller than a single recombination event. Subclonal reporter activation confined to a minor group of mG^+^ cells were observed with or without intratumor spreading in some adenocarcinomas (Figures 6G–6I).

### Constrained tumorigenesis in the thyroid gland of Nkx2.1-CreER^T2^;Braf^CA/+^ mice

**Figure 7:**
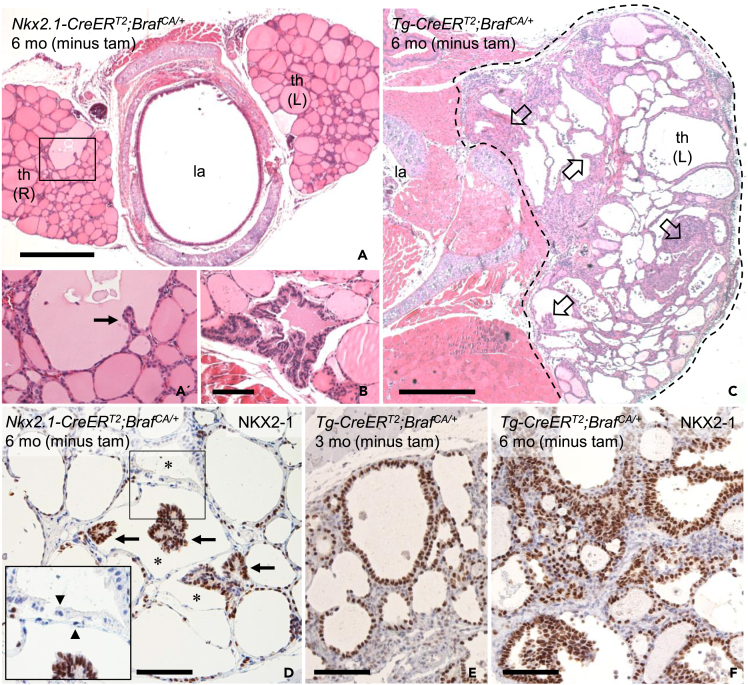
Thyroid tumorigenesis generated by leaky Cre activity from different Cre drivers (*Nkx2-1-CreER*^*T2*^ and *Tg-CreER*^*T2*^) without induced *Braf*^*CA*^ activation Comparison of thyroid phenotype in non-induced *Nkx2.1-CreER*^*T2*^*;Braf*^*CA/+*^ (A, B and D) and *Tg-CreER*^*T2*^*;Braf*^*CA/+*^ (C, E and F) mice. Representative images obtained from 6-month-old (mo) non-induced mutants. (A–C) Thyroid histoarchitecture (HE staining). A′ shows high power of boxed area in A. Open arrows in C indicate multifocality of tumor development. (D–F) NKX2-1 immunohistochemical staining of normal and neoplastic follicles and papillary microcarcinomas. Inset in D shows high power of boxed area. Asterisks indicate follicles with heterogeneous NKX2-1 expression; arrows and arrowheads indicate NKX2-1 positive and negative cells, respectively. th, thyroid lobe (L, left; R, right); la, larynx (lumen). Scale bars: 500 (A, C) and 100 (B, D-F) μm. See also Figure S9.

Because the thyroid of non-induced mutant mice was devoid of macroscopic tumors also at age 6 months (Figures 3D and 3E), we serially sectioned six mutant glands from pole to pole for a thorough histological evaluation. Remarkably, thyroid tissue uniformly displayed a normal follicular architecture apart from occasional enlarged follicles (Figure 7A). Moreover, follicles only rarely showed signs of papillary growth (Figures 7A′ and 7B). This is in marked contrast to the thyroid phenotype of age-matched *Tg-CreER*^*T2*^*;Braf*^*CA/+*^ mice,^4^ which in the absence of tamoxifen induction featured numerous enlarged follicles and multiple tumors spread among follicles with normal size and shape (Figure 7C). The different tumorigenic response, not least evident by lobe size measurements (Figure 3D), suggested that Cre recombinase derived from *Tg-CreER*^*T2*^ had a higher tendency of leaky Cre activity and hence spontaneously trigger recombination than Cre expressed by *Nkx2.1-CreER*^*T2*^ (Figure 3C). On the other hand, because neoplastic follicles are numerous already in 1-month-old *Tg-CreER*^*T2*^*;Braf*^*CA/+*^ mice,^4^ it is likely that a fraction of BRAF mutant cells identified by variant allele sequencing derived from progeny propagation before analysis, countervailing the apparent difference in spontaneous recombination rate.

Next, we investigated immunocytochemically whether NKX2-1 expression in thyroid tumors might be affected similar to early neoplastic lung lesions. Thyroid follicles in general and the few encountered papillary microcarcinomas uniformly consisted of cells with seemingly normal NKX2-1 levels (Figure 7D). However, the encircling epithelium of neoplastic follicles, with or without adjoining papillary tumor profiles, was conspicuously negative of NKX2-1 immunoreactivity (Figure 7D, inset). For comparison, in *Tg-CreER*^*T2*^*;Braf*^*CA/+*^ mice the more numerous neoplastic follicles always showed strong NKX2-1 expression (Figures 7E and 7F). Thus, although thyroid tumor cells eventually regained NKX2-1 expression, downregulation of NKX2-1 was restricted to a subpopulation of cells located close to sites of tumor initiation in *Nkx2.1-CreER*^*T2*^*;Braf*^*CA/+*^ mice. Although we were unable to determine whether NKX2-1 negative thyroid cells were non-mutant or expressed BRAF^V600E^ oncoprotein, these findings are consistent with the hypothesis of cell competition among thyroid follicular cells that might influence early tumor development originating in the same follicle.^4^

Finally, we investigated clonal responsiveness to BRAF activation in the thyroid of *Nkx2.1-CreER*^*T2*^*;Braf*^*CA/+*^;*mTmG* mice. Based on serial sections from 1-, 3-, and 6-month-old mutants (n = 6 for each age), no more than 25 *loci* of reporter gene activation were distinguished per lobe, fewer at earlier timepoints, in non-induced conditions. Thyroid follicles mostly contained single or pairs of mG^+^ cells and there were only minor signs of clonal expansion at age 6 months (Figures S9A and S9B). None of the encountered papillary microcarcinomas were GFP-labeled (data not shown), consistent with a very low incidence of *Braf*^*CA*^ and reporter gene co-activation. By contrast, tamoxifen rapidly induced reporter activation in most thyroid cells except for some enlarged follicles that displayed segmented mG^+^ and mT^+^ labeling of the epithelium (Figures S9C and S9C′). Notably, cells lacking reporter activation were more abundant and comprised several more follicles, although no tumors were observed, 8 weeks after induction (Figure S9D). Expansion of a scattered mT^+^ pool strongly suggested that these cells were refractory to reporter activation, possibly related to diminished NKX2-1 expression by mutant BRAF. A similar heterogeneous response to tamoxifen induction was previously found associated with BRAF^V600E^-induced down-regulation of the Cre driver (thyroglobulin) in *Tg-CreER*^*T2*^*;Braf*^*CA/+*^*;mTmG* mice.^4^

## Discussion

This study provides a new transgenic model for independent investigation of BRAF^V600E^-induced lung and thyroid cancer using the shared lineage-defining transcription factor Nkx2-1 as Cre driver. Unlike other mouse models based on inducible Cre recombinase for conditional targeting, experiments were carried out in non-induced conditions to allow spontaneous oncogene activation – stochastically by leaky Cre – in a minority of cells as documented by direct sequencing of the activated *Braf* mutant allele. Despite initially low percentage of *Braf*^*CA*^ recombination in both organs, multiple lung tumors developed and often progressed to advanced adenocarcinomas, which eventually killed the animals because of locoregional tumor burden, whereas thyroid tumors were extremely rare and limited to small papillary microcarcinomas each confined to a single neoplastic follicle. The different magnitude of tumorigenesis and severity of malignant transformation stand out for adult lung and thyroid tissues whereas the corresponding embryonic progenitor cells showed equally strong responsiveness to oncogenic activation by mutant Braf.

Most murine models of lung cancer use an oncogene-inducing system based on viral transfer of Cre recombinase,^20^ which however results in promiscuous targeting potentially involving any airway cells. Although the inhaled virus titer may be adjusted, it is evident that such widespread activation of MAPK signaling by mutant BRAF leads to a great number of adenomatous tumors that outrival normal lung tissue and shortens animal survival because of respiratory distress rather than by malignant transformation.^1^^,^^2^ Moreover, BRAF^V600E^ alone has a strong tendency to induce senescence of lung tumor cells,^21^ which is inferred as a major explanation to why adenocarcinomas only rarely develop in *Braf*^*CA*^ driven lung tumorigenesis in mice. Indeed, for malignant progression to occur, previous mouse studies indicate that mutant BRAF requires either silencing of a tumor suppressor (e.g., TP53) or co-activation of another oncogenic pathway (e.g., PI3K).^22^^,^^23^^,^^24^^,^^25^ *Nkx2.1-CreER*^*T2*^*;Braf*^*CA*^ mice represent an alternative and conceptually more suitable lung cancer model by which BRAF^V600E^-driven tumorigenesis can be monitored stagewise from benign nodules to invasive carcinomas without additional genetic manipulations other than the *Nkx2-1* null heterozygous background genotype.

Uniform expression of SPC in the vast majority of lung tumors strongly suggested that oncogenic activation predominantly originated in AT2 pneumocytes. This finding is consistent with previous reports that SPC^+^ lung cells more efficiently develop tumors than CC10^+^ cells following targeted expression of mutant Kras.^26^ Because all lung cells originally derive from the lung bud and transiently express NKX2-1, and distal bronchiolar cells maintain NKX2-1 expression until adulthood, findings of tumorigenesis taking place preferentially in the alveolar compartment support previous notions that AT2 cells are specifically vulnerable to activating mutations of the MAPK signaling pathway.^27^ From a clinical point of view, the current lung tumor model also mimics the typical features of Braf mutation-driven NSCLC in humans.^10^^,^^11^

We recently reported that multifocal PTC readily developed by using a similar approach for stochastic BRAF^V600E^ expression with thyroglobulin as Cre driver.^4^ The much lower susceptibility of thyroid tumor initiation in the current *Nkx2.1CreER*^*T2*^-based model might relate to the documented difference in prevalence of spontaneous recombination comparing the two Cre drivers. In non-induced *Nkx2.1-CreER*^*T2*^*;Braf*^*CA*^ mice, it was also a trend difference in cell numbers carrying the activated mutant *Braf* allele between lung and thyroid tissues. However, considering the early onset of tumorigenesis, it is conceivable that propagation of BRAF mutant progeny in the lungs prior to sequencing explains most if not all the monitored organ differences in active *Braf* allele content. It is noteworthy that potentially tumorigenic cells with active Braf comprised less than 1% of the total cell number in both tissues. Altogether, these observations strongly favor the existence of local tissue factors that influence the outcome of oncogenic activation in an organ-specific fashion.

Mice expressing *Nkx2.1-CreER*^*T2*^ for conditional targeting are *Nkx2-1* heterozygous and potentially haploinsufficient.^12^ We found here that Nkx2-1 conspicuously down-regulates in early neoplastic lesions in both lung and thyroid of non-induced mutants, suggesting that both *Nkx2-1* alleles are required for stable expression in a situation of constitutive activation of the MAPK signaling pathway. This is consistent with observations that oncogenic Ras through Raf dose-dependently represses Nkx2-1 both in terms of protein expression and functional activity in established thyroid cell lines.^28^^,^^29^ Although tumor cells re-expressed Nkx2-1 in more advanced tumor stages, it is thus possible that monoallelic loss of *Nkx2-1* influences differently BRAF mutant lung and thyroid cells during early tumor development. Previous reports indicate that haploinsufficiency for *Nkx2-1* promotes development of KRAS-induced mucinous lung adenocarcinoma,^30^ and that re-expressed Nkx2-1 constrains progression and metastasis of poorly differentiated (NKX2-1 negative) *Kras* ^*LSL-G12D/+*^*;p53*^*flox/flox*^ lung cancer cells.^31^ Loss of Nkx2-1 may even push the differentiation program toward a gastric lineage in both normal and neoplastic lung cells.^32^^,^^33^ More recent studies infer a tuning role of Nkx2-1 in the homeostatic feedback mechanism that dampens MAPK signaling and prevents malignant progression of RAS-driven lung tumors.^34^ On the other hand, *NKX2-1* is amplified and a suggested oncogene in human lung cancer.^35^^,^^36^^,^^37^ Recalling that *Braf* mutation as a single oncogenic event confers growth-arrest and senescence of *Nkx2-1*^*+/+*^ mouse lung tumor cells at a premalignant stage,^1^ our study is the first demonstrating that reduced Nkx2-1 expression might promote BRAF^V600E^-induced lung tumor development and progression to malignancy.

Nkx2-1 is required for both embryonic thyroid differentiation^38^^,^^39^ and thyroid developmental growth.^40^ From previous studies in *T/ebp(fl/fl);TPO-Cre* conditional hypomorphic mice it is also known that both *Nkx2-1* alleles are required for maintenance of thyroid follicular architecture and the ability of follicles to regenerate.^41^ The present study raises the possibility that biallelic transcriptional activity of Nkx2-1 might exert a permissive role also for oncogenic activation and neoplastic growth in the adult thyroid gland. A related issue concerns TSH-stimulated thyroid cell proliferation and previous notions that TSH promotes BRAF-driven tumorigenesis in mouse models.^42^ TSH appears important already for tumor initiation by counteracting oncogene-induced senescence in thyroid cells.^43^ The fact that *Nkx2-1* heterozygous mice have functionally impaired thyroid hormone synthesis and suffers from subclinical hypothyroidism with elevated TSH levels^44^ would argue, contrary to present findings, that *Nkx2-1* haploinsufficiency rather would facilitate thyroid tumor development. On the other hand, reduced *Tshr* expression levels^45^ and impaired growth response to TSH^46^ indicate that *Nkx2-1*^*+/−*^ thyroid cells have impaired TSH receptor signaling, which thus might contribute to refractoriness to oncogenic activation in the thyroid of *Nkx2.1-CreER*^*T2*^*;Braf*^*CA*^ mice.

There are other cellular mechanisms not directly involved in mitogenic signaling pathways that might influence the outcome of *Braf* mutation. In a recent study comparing immediate effects of ubiquitous BRAF^V600E^ expression in mice,^47^ a DNA damage response was evident in several organs including the lungs but not in the thyroid. Although these organ differences were not further investigated, it is possible that cells’ ability to cope with DNA damage on oncogene activation might differentially influence tumorigenesis in lung and thyroid. Tissue specificity to oncogenic mutations might also concern tumor clonality. In the present study, lineage tracing revealed that spontaneously developed lung tumors in *Nkx2.1-CreER*^*T2*^*;Braf*^*CA/+*^ mice had a monoclonal origin. It was not possible to simultaneously trace tumor clonality in the thyroid presumably because of the limited number and poor growth of neoplastic lesions. However, in the thyroid of *Tg-CreER*^*T2*^*;Braf*^*CA/+*^ mutants it is evident that sporadic tumor development is an oligoclonal trait based on recruitment of multiple clones at an early stage, whereas single mutant cells surrounded by non-mutant follicular cells are not tumorigenic.^4^ Inability of adult thyroid cells to initiate and propagate clonal growth on oncogenic activation may thus involve cell competition from adjacent non-mutant cells in the same follicle. Impaired thyroid tumorigenesis after induced *Braf*^*CA*^ activation, another major difference to mouse lung tissue, must nevertheless involve a different mechanism.

In conclusion, we have identified and characterized a Cre/loxP-based mouse model that allows independent investigation of sporadic lung and thyroid cancer that largely mimic the features of BRAF-driven NSCLC and PTC in humans. The dual lung-thyroid cancer model will be useful to decipher and further characterize tissue-specific factors that either promote or constrain tumor development and progression to advanced and clinically significant malignancies. It may also be instrumental in testing new targeted drug therapies designed to interfere with different tumor stages.

### Limitations of the study

Inherent to the *Cre-ER*^*T2*^ fusion gene construct, conditional targeting simultaneously abolished function of the Cre driver (*Nkx2-1*). It was therefore not possible to exert control experiments of oncogenic effects with remained function of both *Nkx2-1* alleles. Suggested impact of *Nkx2-1* haploinsufficiency on BRAF^V600E^-induced tumor phenotypes relies on circumstantial evidence including observations of reduced NKX2-1 expression levels in early neoplastic lesions. On similar grounds, because constitutive *Braf*^*CA*^ activation in embryonic tissues occurred with preserved function of both wildtype *Nkx2-1* alleles, it remains to be elucidated whether thyroid and lung progenitors might respond differently to mutant Braf in a *Nkx2-1* heterozygous background. Presently, there are no other suitable Cre drivers that specifically targets Nkx2-1-lineage cells and would be instrumental to further address *in vivo* the potential role of *Nkx2-1* gene dosage in sporadic tumor development in thyroid and lung.

## STAR★Methods

### Key resources table

**Table:** 

REAGENT or RESOURCE	SOURCE	IDENTIFIER
**Antibodies**		

Rabbit polyclonal anti-NKX2-1	BioPat	PA0100/1; RRID:AB_2313674
Rabbit polyclonal anti-PAX8	Proteintech	10336-1-AP/1; RRID:AB_2236705
Rat polyclonal anti-CDH1 (ECCD-2)	Thermo Fisher	13-1900; RRID:AB_86571
Rabbit polyclonal anti-SPC (proSP-C)	Sigma-Aldrich	AB3786; RRID:AB_91588
Rabbit polyclonal anti-CC10	Millipore	07-623; RRID:AB_310759

**Bacterial and virus strains**		

Ad5CMVCre *Cre* adenovirus	Viral Vector Core Facility, University of Iowa	VVC-U of Iowa-5

**Chemicals, peptides, and recombinant proteins**		

Tamoxifen	Sigma-Aldrich	T5648
Q5 HotStart Polymerase	New England BioLabs	M0491S
Platinum™ SuperFi II DNA Polymerase	Thermo Fisher	12361010

**Critical commercial assays**		

PT Link	Dacocytomation	Ref. PT10130
Dako EnVision System	Dacocytomation	Ref. K8004, K8007, K8000, K8006, K8009, K4003, K3468
Rat Impress System	Vector laboratories	MP-7444
OCT Tissue-Tek	Sakura	45830
DAPI Nuclear Stain	Sigma-Aldrich	D8417
Qiamp genomic DNA extraction kit	Qiagen	51404
Agencourt AMPure XP system	Beckman Coulter	A63880

**Deposited data**		

Sequencing data	This paper	NCBI Sequence Read Archive (SRA): PRJNA880479

**Experimental models: Organisms/strains**		

Mouse: Braf^CA^	The Jackson Laboratory	JAX Stock: #017837; RRID:IMSR_JAX:017837
Mouse: Nkx2.1-Cre	The Jackson Laboratory	JAX Stock: #008661; RRID:IMSR_JAX:008661
Mouse: Nkx2.1-CreER^T2^	The Jackson Laboratory	JAX Stock: #014552; RRID:IMSR_JAX:014552
Mouse: ROSA^mT/mG^	The Jackson Laboratory	JAX Stock: #007676; RRID:IMSR_JAX:007676
Mouse: Tg-CreER^T2^	The Jackson Laboratory	JAX Stock: #030676; RRID:IMSR_JAX:030676

**Oligonucleotides**		

Braf forward barcoding primer: GTGAC TGGAGTTCAGACGTGTGCTCTTCCGATC TTGAGTATTTTTGTGGCAACTGC;Braf reverse barcoding primer, GGACACTC TTTCCCTACACGACGCTCTTCCGATCT NNNNNNNNNNNNATGGGAAAGAGT GTCCCTCTGCTGGGAAAGCGG	This paper	N/A

**Software and algorithms**		

OuPath	Open source	Bioimage analysis written with JavaFX
Prism 9	GraphPad Software	Version 9.5.0 (525)
FastX toolkit versjon 0.0.13	http://hannonlab.cshl.edu/fastx_toolkit/index.html	N/A
Bwa mem version 0.7.17	https://bio-bwa.sourceforge.net/	N/A
NIS Elements Imaging. Software	Nikon	Version 4.4

**Other**		

SiMSen-Seq	Ståhlberg et al.^15^	N/A
Fragment Analyzer	Agilent	M5311AA
MiniSeq Instrument	Illumina	N/A
Olympus BX45TX	Olympus	SN.0C07201
Zeiss Axioscop2 plus	Zeiss	ID/SIPnr 040-020853
Tissue Lyser II	Qiagen	85300

### Resource availability

#### Lead contact

Further information and requests for resources and reagents should be directed to and will be fulfilled by the lead contact, Mikael Nilsson (mikael.nilsson@gu.se).

#### Materials availability

All mouse lines employed in this study are available at Jackson Laboratory.

This study did not generate new unique reagents.

### Experimental model and study participant details

Mice heterozygous for mutant *Braf* (*Braf*^*CA/+*^) originally developed for BRAF^V600E^ expression in the lung^1^ (JAX Stock: #017837; B6.129P2(Cg)-*Braf*^*tm1Mmcm*^/J; Jackson Laboratory) were crossed with mice with different driver constructs of Cre recombinase: *Nkx2-1-Cre*^48^ (JAX Stock: #008661: C57BL/6J-Tg(Nkx2-1-cre)2Sand/J; Jackson), *Nkx2.1-CreER*^*T2*^ (JAX stock #014552: *Nkx2-1*^*tm1.1(cre/ERT2)Zjh*^; Jackson) and *Tg-CreER*^*T2*^ (JAX Stock: #030676; C57BL/6N-Tg(Tg-cre/ERT2)1Kero/J; Jackson). The *Nkx2.1-CreER*^*T2*^ fusion gene was designed to abolish *Nkx2-1* transcriptional activity.^12^ Notably, homozygous inactivation of *Nkx2-1* causes athyreosis and defective lung development^5^ whereas haploinsufficiency confers a mild thyroid phenotype in mice.^44^ *Tg-CreER*^*T2*^ used the *thyroglobulin* promoter as Cre driver and therefore expressed in thyroid only.^4^ For clonal tracing, all *Cre* lines were additionally crossed with the *mTmG* double fluorescent reporter^49^ (JAX Stock: #007676; B6.129(Cg)-*Gt(ROSA)26Sor*^*tm4(ACTB-tdTomato,-EGFP)Luo*^/J; Jackson). Strains were backcrossed with C57BL/6J mice at least 10 generations before recombination experiments. Males and females were equally represented in experiments; there was no comparison of sex-biased outcome in this study. Investigated developmental stages were: *i*) Embryonic days (E) E10.5–18.5, *ii*) Postnatally (P) P0, P10 and P30, and *iii*) Adults at 1, 3, 6 and 9 months of age. Ear punch biopsies were sampled for genotyping with PCR. Tamoxifen (Sigma-Aldrich) dissolved in sunflower oil (10 mg/mL) was injected intraperitoneally (50 μL) daily ×4 for CreER^T2^ induction. *Braf*^CA/+^ mice were subjected to inhalation of a Cre adenoviral vector (Viral Vector Core Facility, University of Iowa) administered as a calcium phosphate precipitate (5×10^7^ plaque-forming units (PFU)) under general anesthesia, as described.^1^ Survival endpoints of mutant mice were primarily based on lung tumor burden that required euthanasia due to compromised breathing. Animal experiments were approved by the regional ethical committee (Dnr 5.8.18–03925/2018) according to European standards and national regulations provided by the Swedish Agriculture Agency.

### Method details

#### Wildtype and mutant braf allele sequencing and quantification

Thyroid and lung tissue samples (lobes or part of lobes) were removed of gland capsule/visceral pleura and homogenized by TissueLyser II (Qiagen) followed by preparation of DNA using the Qiamp genomic DNA extraction kit (Qiagen) according to manufacturer instructions. SiMSen-Seq was performed as previously described.^15^ Briefly, unique molecular identifiers were attached to target DNA molecules in a 10 μL barcoding PCR consisting of 0.05 U Platinum SuperFi I DNA polymerase, 1x SuperFi Buffer (both Thermo Fisher Scientific), 0.2 mM dNTP (Sigma-Aldrich), 0.5 M L-Carnitine inner salt (Sigma-Aldrich), 40 nM of each barcoding primer (Ultramer, IDT), and 100 ng DNA. The barcoding primer pair flanked the insertion site of the *Braf* gene and the following sequences were used: forward barcoding primer, GTGACTGGAGTTCAGACGTGTGCTCTTCCGATCTTGAGTATTTTTGTGGCAACTGC; reverse barcoding primer, GGACACTCTTTCCCTACACGACGCTCTTCCGATCTNNNNNNNNNNNNATGGGAAAGAGTGTCCCTCTGCTGGGAAAGCGG. The thermal program was: 30 s at 98°C, followed by 3 cycles of amplification (98°C for 10 s, 62°C for 6 min, 72°C for 30 s), 15 min at 65°C, and 15 min at 95°C. One-third of the barcoding reaction was amplified in a second 40 μL adapter PCR containing 1x Q5 HotStart Polymerase (New England BioLabs) and 400 nM Illumina adapter primers (IDT, desalted). The thermal program was: 98°C for 3 min, followed by 28 cycles of amplification (98°C for 10 s, 80°C for 1 s, 72°C for 30 s, 76°C for 30 s). Libraries were purified using the Agencourt AMPure XP system (Beckman Coulter) and a 1:1 beads-to-sample ratio, according to manufacturer instructions. Prior to sequencing, library products were assessed on a Fragment Analyzer (Agilent) to confirm purity and size distribution. Sequencing was performed on a MiniSeq instrument (Illumina), according to the manufacturer instructions.

For sequencing data analysis adapters were first trimmed from raw fastq files using cutadapt (version 3.4) and default parameters. Poor-quality reads were removed using fastq_quality_filter from the FastX toolkit (version 0.0.13) with settings ‘-q 20 -Q33 -p 100’. Trimmed and filtered reads were aligned to a custom reference genome using bwa mem (version 0.7.17) and processed with a custom python pipeline matching each read to either the wildtype, inactive mutant or active mutant *Braf* sequences. Reads were grouped into barcode families if there were ≥3 reads with the same unique molecular identifiers to eliminate PCR duplicates and enable accurate quantification of cell numbers. A small amount of noise remained after UMI correction. Hence, ≤6 barcode families were considered as background noise.

#### Immunostaining and microscopy

After sacrifice, *in situ* thyroids and excised lungs were photographed together with a ruler for size-measurements with an iPhone in-built camera. Organs were immersion-fixed in 4% paraformaldehyde and further processed for routine hematoxylin–eosin (HE) staining of paraffin sections and immunohistochemical staining of deparaffinized sections subjected to epitope retrieval by PT Link (Dacocytomation) and quenching of endogenous peroxidase activity. Immunostaining was optimized with the Dako EnVision system (Dacocytomation) for antibodies against (catalog number/titer/company): NKX2-1 (PA0100/1:1000; BioPat) and PAX8 (10336-1-AP/1:2000; Proteintech); and with the Rat Impress system (Vector Laboratories) for: anti-E-cadherin/CDH1 (13–1900:4000; Thermo Fisher), surfactant protein C/SPC (AB3786/1:1600; Millipore), and club cell protein 10/CC10 (07-623/1:800; Millipore). Sections were viewed and imaged in an Olympus BX45TF microscope equipped with a Nikon DS-U2 camera. For evaluation of *mTmG* reporter activation, paraformaldehyde-fixed tissue samples were incubated in 30% sucrose overnight, embedded in OCT Tissue-Tek (Sakura) and stored at −80°C until analysis. Cryosections were collected on Super Frost glass slides (Vector) and counter-stained with DAPI nuclear stain (Sigma-Aldrich) before mounting with fluorescence mounting medium (Dakocytomation). Fluorescent mT^+^ and mG^+^ cells were analyzed in a Zeiss Axioskop2 plus microscope equipped with a Nikon DS-Qi1Mc camera. Image acquisition and processing used the NIS Elements Imaging Software.

### Quantification and statistical analysis

#### Morphometry

Thyroid lobe volumes were estimated by using the standard formula for ellipsoids, $=Height\;x\;Width\;x\;Depth\;x\;\frac{\pi }{6}$ , as reported,^50^ based on lobe diameters measured with a digital slide gauge: longitudinally from pole to pole (height) and transversely from medial to lateral margins (width); the transverse diameter was also used to estimate lobe size dorsoventrally (depth). Overall lung tumor burden was estimated by calculating the tumor area per total lung area in HE-stained sections for 3 standardized sections levels per specimen using QuPath software. Separate lesions in the same sections were classified by histological characteristics as i) hyperplasia, ii) adenoma and iii) adenocarcinoma.

#### Statistics

Statistical analyses were made using Prism 9 for Mac Os Monterey version 12.2.1 (GraphPad Software, Inc.). Analyses included unpaired t-tests and one-way ANOVA tests assuming Gaussian distribution (after testing for normality) with graph error bars displaying mean ± standard error of the mean (SEM). For Kaplan-Meier survival analysis, Mantel-Cox Chi square test was performed. A p value below 0.05 (∗<0,0332, ∗∗<0,0021, ∗∗∗<0,0002, ∗∗∗∗<0,0001) was considered statistically significant. Numbers of mice/specimens are indicated in figures and figure legends.

## Data Availability

• Raw sequencing data have been uploaded to the NCBI Sequence Read Archive (SRA) under accession PRJNA880479 (http://www.ncbi.nlm.nih.gov/bioproject/880479). Adjective data reported in this paper will be shared by the lead contact upon request. • This paper does not report original code. • Any additional information required to reanalyze the data reported in this paper is available from the lead contact upon request.
