# Supplementary material for: Tissue specificity of oncogenic BRAF targeted to lung and thyroid through a shared lineage factor
Source: iScience. 2023 Jun 8;26(7):107071. doi: 10.1016/j.isci.2023.107071 (PMC10391731; doi:10.1016/j.isci.2023.107071)
Supplement: Document S1. Figures S1–S9 and Table S1 [file mmc1.pdf]

iScience, Volume *26*

Supplemental information

Tissue specificity of oncogenic BRAF targeted to lung and thyroid through a shared lineage factor

Elin Schoultz, Shawn Liang, Therese Carlsson, Stefan Filges, Anders Ståhlberg, Henrik Fagman, Clotilde Wiel, Volkan Sayin, and Mikael Nilsson

Table S1. Survival of Braf mutant mice depending on method of Cre activation, Related to Figure 3.

A. Non-induced vs adeno-Cre: comparison of survival curves[1]

Chi square	8,817	
df	1	
P value	0,0030	
P value summary	**	
Are the survival curves sig different?	Yes	
Gehan-Breslow-Wilcoxon test		
Chi square	9,580	
df	1	
P value	0,0020	
P value summary	**	
Are the survival curves sig different?	Yes	
Median survival		
Non-induced	24,00	
adeno-Cre	19,50	
Ratio (and its reciprocal)	1,231	0,8125
95% CI of ratio	0,5935 to 2,552	0,3918 to 1,685
Hazard Ratio (Mantel-Haenszel)	A/C	C/A
Ratio (and its reciprocal)	0,1933	5,174
95% CI of ratio	0,06530 to 0,5719	1,749 to 15,31
Hazard Ratio (logrank)	A/C	C/A
Ratio (and its reciprocal)	0,3843	2,602
95% CI of ratio	0,1449 to 1,019	0,9812 to 6,901

B. Non-induced mouse survival[2]

Weeks alive	No. animals
12	2
16	1
20	1
22	2
23	3
24	10
25	2
26	1
32	1
36	2

[1]Extended data calculations complimentary to survival graphs of non-induced *Nkx2.1-CreER^{T2};Braf$^{CA/+}$* mice (n=25) and induced *Braf$^{CA/+}$* mutants exposed to an adenoviral Cre vector (n=15), as shown in Fig. 4D.
[2]Survival of individual *Nkx2.1-CreER^{T2};Braf$^{CA/+}$* mice in the non-induced cohort.

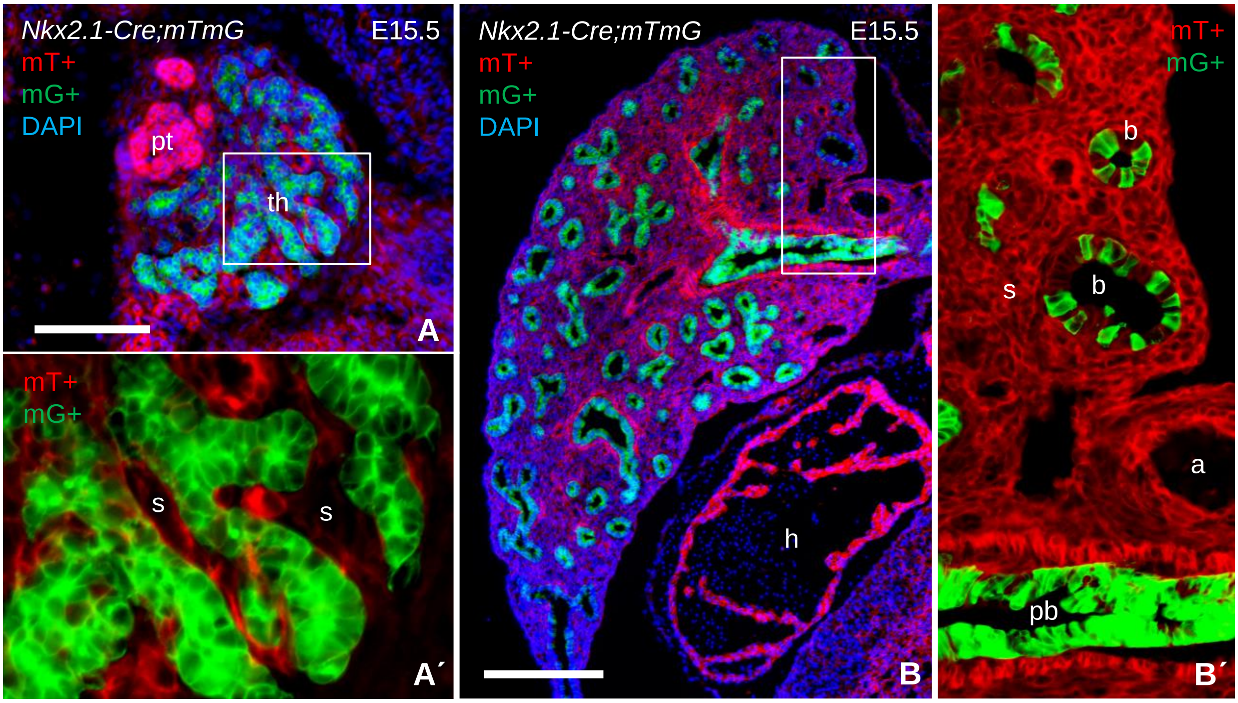

Fig. S1. Reporter gene activation targeted to NKX2-1 expressing cells in embryonic thyroid and lung tissues, Related to Figure 2. Images from serial sections of a *Nkx2.1-Cre;mTmG* mouse embryo collected at embryonic day 15.5 (E15.5). Switching from red (mTomato) to green (GFP) fluorescence occurs upon activation of the dual fluorescent reporter, as indicated by mT^+ (by default) and mG^+ (Cre-mediated) labeling of cells. **A, A´**) Right thyroid lobe, overview (A) and close-up (A´, boxed area in A), indicate homogeneous *mTmG* activation in thyroid progenitor cells during branching morphogenesis. **B, B´**) Right lung, overview (A) and close-up (A´, boxed area in A), indicate *mTmG* activation predominantly in the developing bronchial tree. Note mosaic mT^+ labeling consistent with heterogeneous Cre-mediated recombination in this embryonic tissue. DAPI nuclear stain omitted in high power images for impoved clarity. th, thyroid lobe; pt, para-thyroid; h, heart (rigth atrium); pb, principal bronchus; b, bronchi; s, stroma. Bars: 500 (B) and 100 (A) µm.

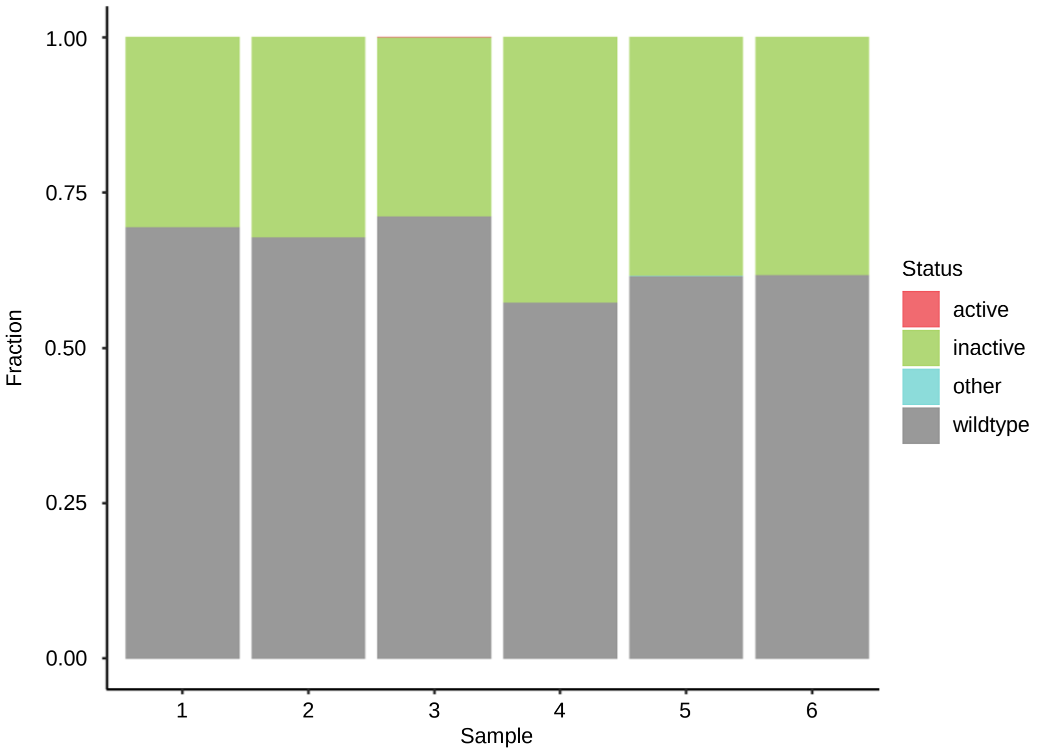

Fig. S2. Lack of spontaneous *Braf*^{CA} activation in newborn *Nkx2.1-CreER*^{T2};*Braf*$^{CA/+}$ mice, Related to Figure 3. Lung samples (n=6) collected immediately after birth from offspring to heterozygous mice were subjected to quantification of inactive and activated *Braf* mutant alleles and the wildtype *Braf* counterpart by SimSenSeq analysis, as detailed in STAR Methods. In absence of tamoxifen, there were no measurable signs of spontaneous Cre-mediated *Braf*^{CA} activation at P0, comfirming that sporadic lung tumorigenesis in non-induced conditions as described in the present paper is entirely a postnatal phenomenon. Sample genotypes: *Braf*$^{CA/+}$ (1-3) and *Nkx2.1-CreER*^{T2};*Braf*$^{CA/+}$ (4-6).

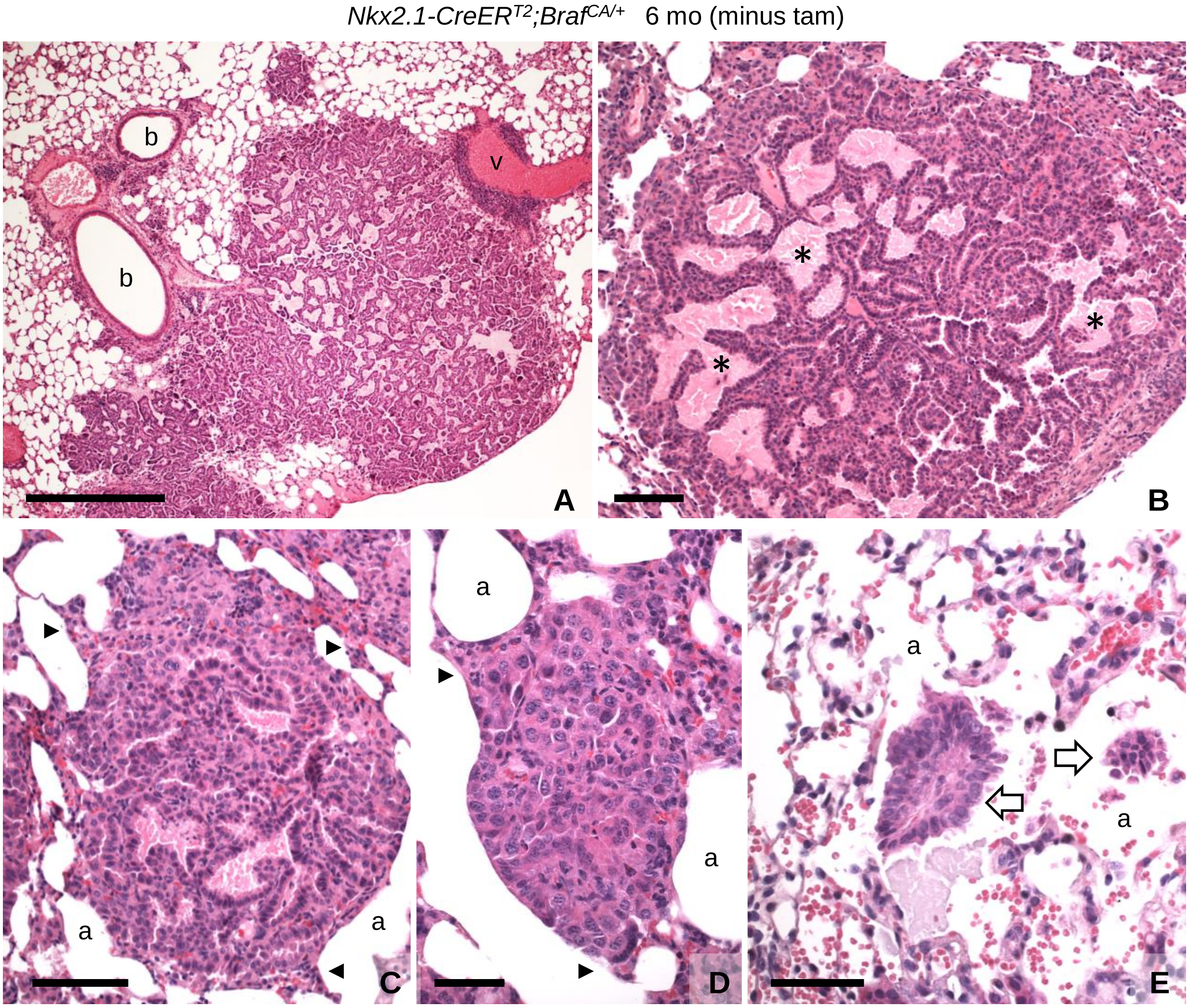

Fig. S3. Advanced lung tumor stages of in *Nkx2.1-CreER^{T2};Braf$^{CA/+}$* mice, Related to Figure 4. Representative images of HE-stained paraffin lung tissue sections from 6 months old non-induced mutants. **A**) Large adenocarcinoma with papillary growth pattern. **B**) Papillary adenocarcinoma with accumulation of exudate in the irregular lumen (asterisks). **C-E**) Invasive tumor portions that infiltrate the interalveoalar (arrrowheads) and intraalveolar (open arrows) space. Note luminal hemorrage in E. b, bronchi; v, vessel; a, alveoli. Bars: 500 (A), 100 (B and C) and 50 (D and E) μm.

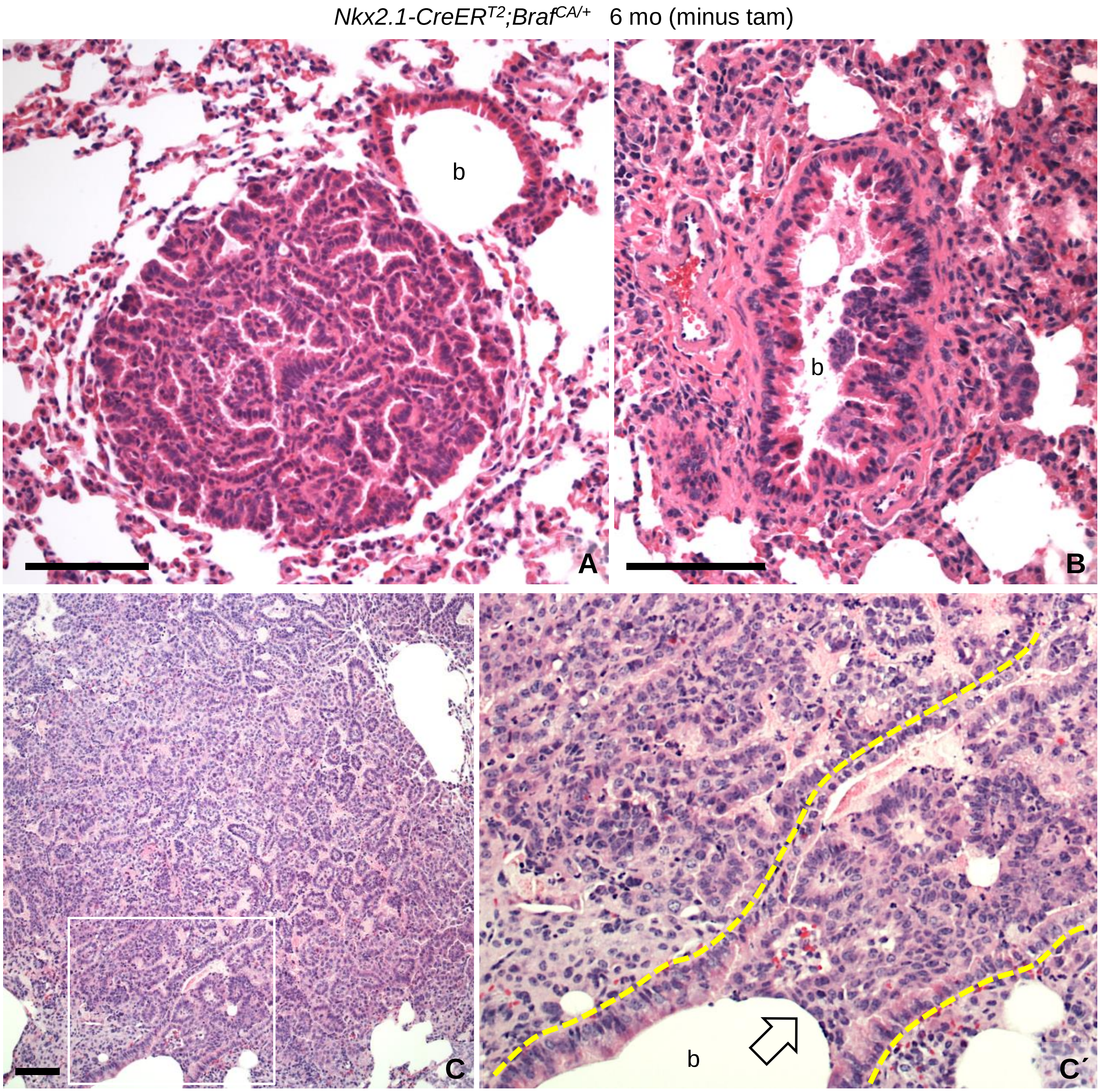

Fig. S4. Tumor–bronchial relationship, Related to Figure 4. Examples of BRAF-induced lung tumors associated with the bronchial tree in *Nkx2.1-CreER*^{T2};*Braf*$^{CA/+}$ mice following spontaneous *Braf*^{CA} activation. **A**) Adenoma located close to but not directly emerging from a bronchus. **B**) Hyperplastic growth of the epithelium in a bronchial segment. **C**) Large adenocarcinoma enclosing a bronchus. C´ shows high power of boxed area in C with the bronchial epithelium outlined (yellow). Open arrow indicates edge of tumor tissue that infiltrate the bronchial lumen. b, bronchi; v, vessel; a, alveoli. Bars: 100 μm.

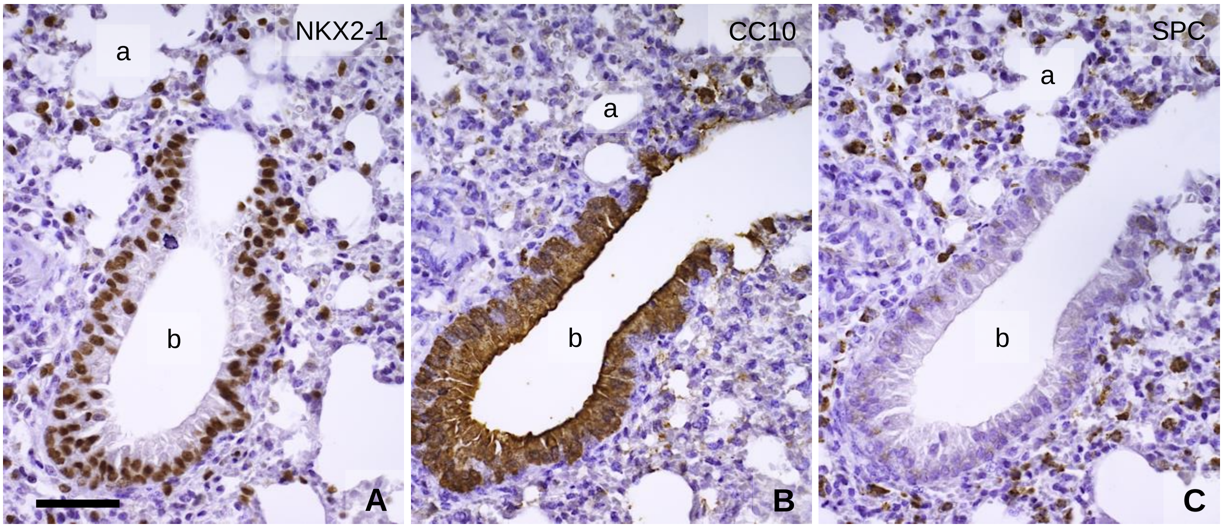

Fig. S5. Lung biomarkers distinguishing brochiolar and alveolar cells, Related to Figure 5. Immunohistochemical images of NKX2-1, club cell protein 10 (CC10) and surfactant protein C (SPC) expression in wildtype (wt) mouse peripheral lung tissue (from parallel sections) comfirming potential use as biomarkers of tumor cell origin. **A**) NKX2-1 antibody labels both cell types. **B**) CC10 antibody exclusively labels bronchiolar epithelium. **C**) SPC antibody exclusively labels alveolar type 2 (AT2) cells. Bar: 50 µm.

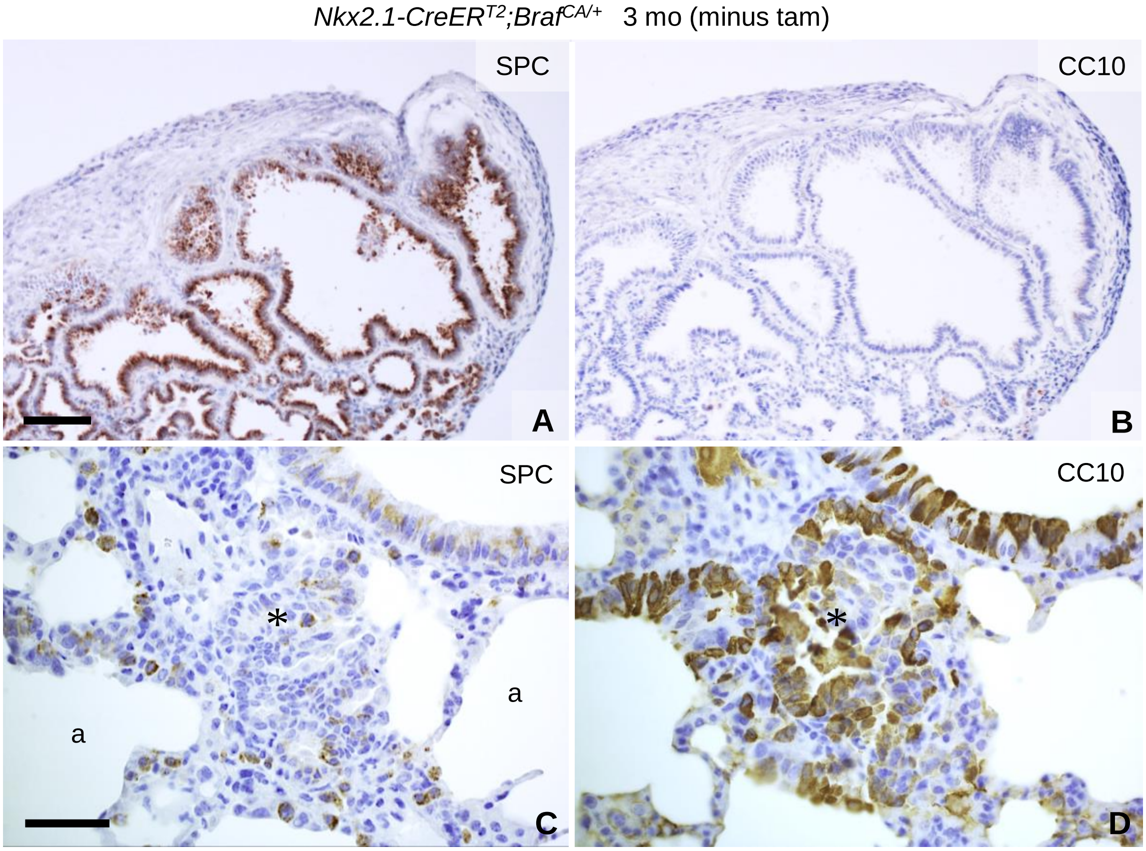

Fig. S6. Mutually exclusive expression of SPC and CC10 in lung tumor cells, Related to Figure 5. Immunostaining of lung tissue sections for surfactant protein C (SPC) and club cell protein 10 (CC10) in non-induced *Nkx2.1-CreER^{T2};Braf$^{CA/+}$* mice. **A, B**) Large and partly cystic SPC^{+}/CC10$^{–}$ lung adenocarcinoma. **C, D**) Lung neoplasia consisting predominantly of CC10^{+} cells. Note heterogeneous CC10 immunostaining of tumor cell cluster that are essentially negative for SPC. (asterisks). This is one of only two lung tumors scored SPC^{-}/CC10^{+} in all examined specimens, as indicated in Fig. 5G. b, bronchus; a, alveole. Bars: 100 (A, B) and 50 (C, D) μm.

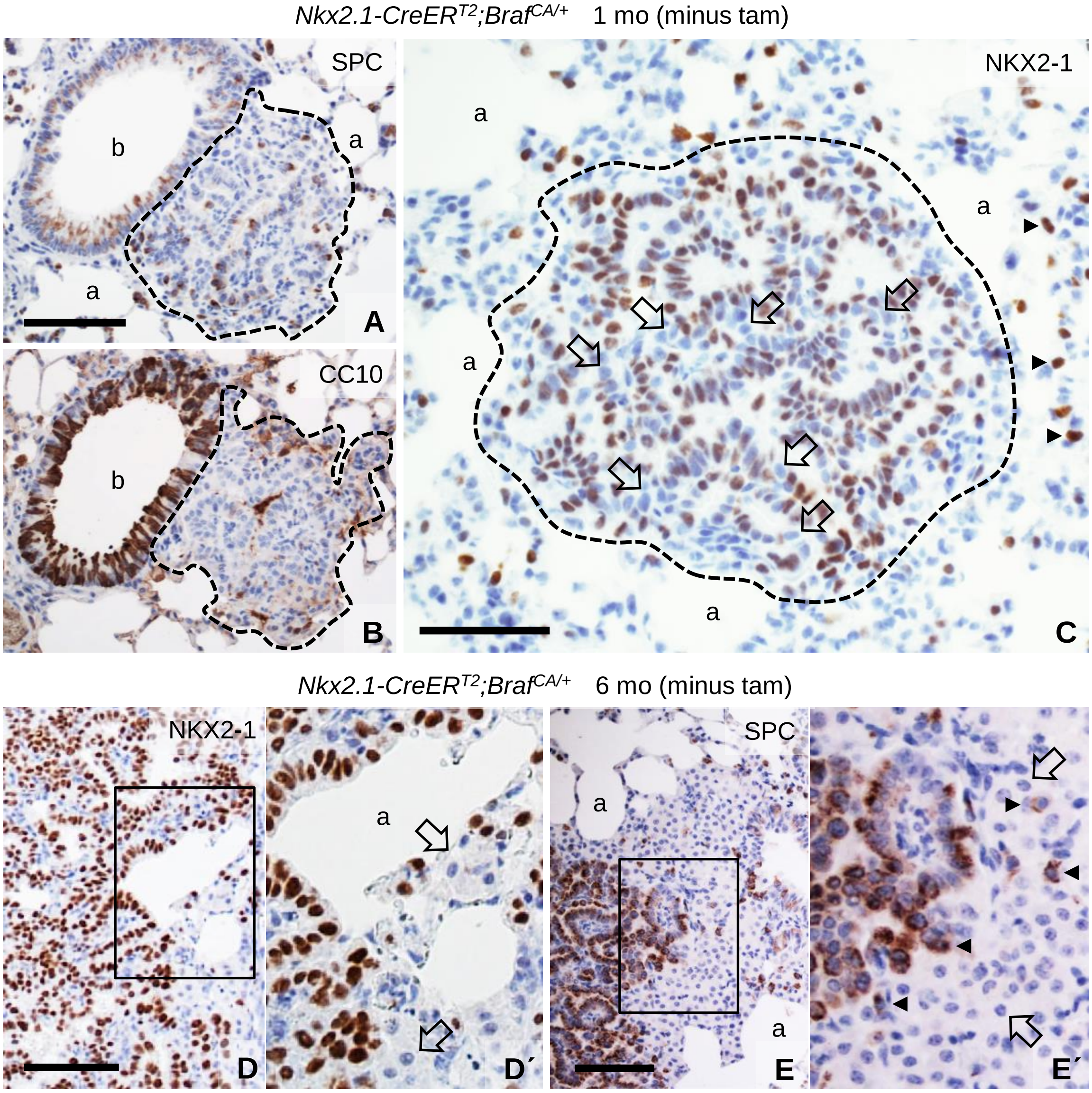

Fig. S7. Downregulation of NKX2-1 and SPC in early lung tumor development and tumor progression, Related to Figure 5. Lung tissue sections immunostained for surfactant protein C (SPC), club cell protein 10 (CC10) and NKX2-1 in non-induced *Nkx2.1-CreER*^{T2}*;Braf*$^{CA/+}$ mice. **A, B**) Small CC10 negative adenoma with weak SPC staining comprising few, scattered SPC^{+} cells (encircled). Note aberrant weak SPC staining in adjacent bronchiolar epithelium (a common finding in mutants); CC10 in tumor centre is artefact (located interstitially). **C**) Heterogeneous NKX2-1 expression in small lung adenoma (encircled). Open arrows indicate tumors cells with reduced or absent nuclear immunoreactivity for NKX2-1; arrowheads indicate alveolar cells with strong NKX2-1 staining. **D**) Invasive front of NKX2-1^{+} lung adenocarcinoma. D´ shows high power of boxed area in D. Open arrows indicate NKX2-1 negative cells at tumor border. **E**) Invasive front of SPC^{+} lung adenocarcinoma. E´ shows high power of boxed area in E. Open arrows indicate SPC negative cells at tumor border; arrowheads indicate dissociated SPC^{+} cells in the same location. b, bronchus; a, alveolus. Bars: 100 (A, B, D, E) and 50 (C) µm.

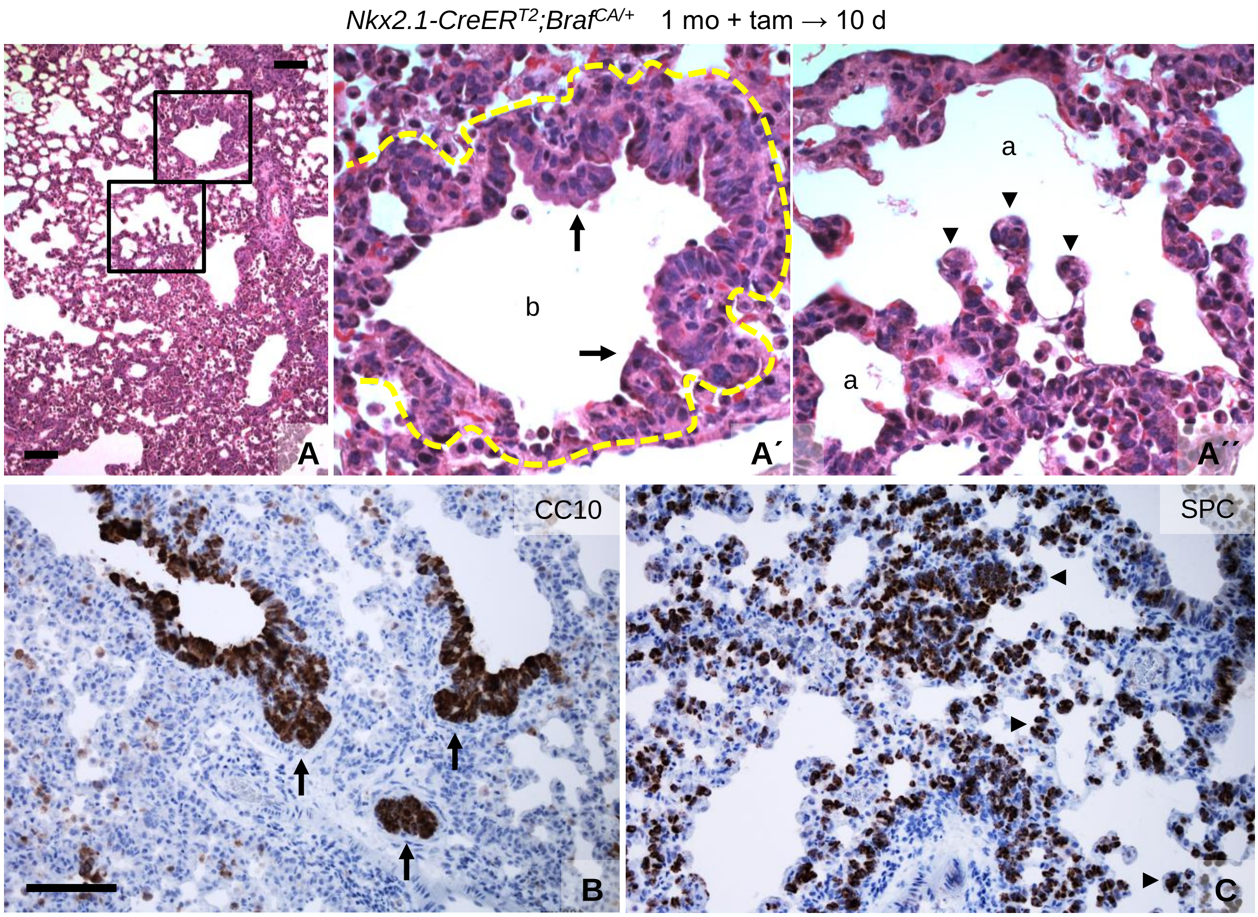

Fig. S8. Proliferative response to induced *Braf*^{CA} activation in adult lung NKX2-1^{+} cells, Related to Figure 5. Young adult *Nkx2.1-CreER*^{T2}*;Braf*$^{CA/+}$ mice were injected with tamoxifen (x4) and sacrificed already after 10 days for lung tissue examination with HE and IHC stainings. **A**) General lung hyperplasia with increased cellularity in all compartments. A´ and A´´ show high power of boxed areas with hyperplasia of bronchial epithelium (arrows in A´, folded epithelium encircled) and alveoli (arrowheads in A´´).
B) Pathological expansion of CC10^{+} bronchiolar epithelium into surrounding tissue (arrows). **C**) Massive increase of SPC+ cells on expense of the alveolar space (arrowheads). b, bronchi; a, alveoli. Bars: 100 μm.

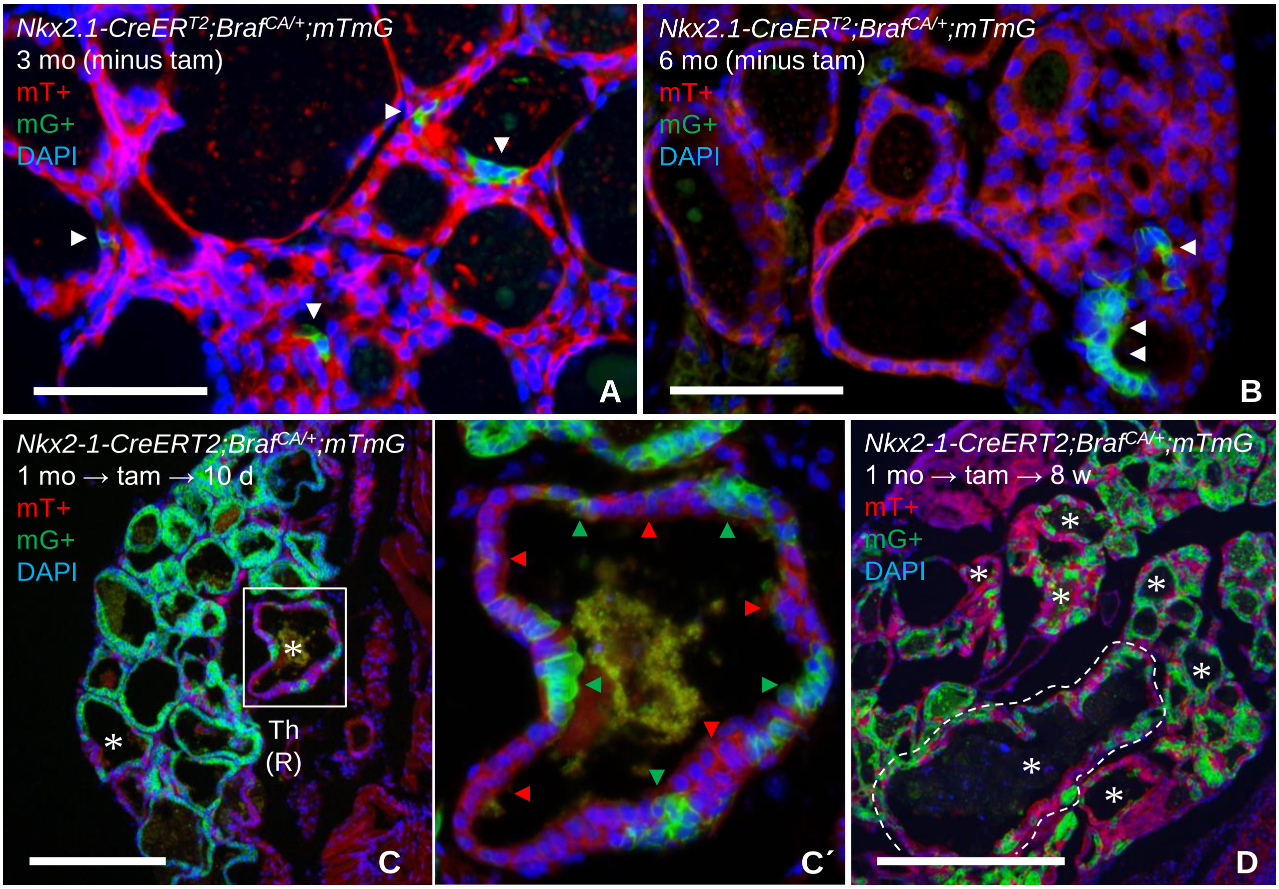

Fig. S9. Clonal tracing in the thyroid of *Nkx2.1-CreRER^{T2};Braf^{CA};mTmG* mice, Related to Figure 7. Experiments were primarily conduced to demonstrate the basal rate of spontaneous recombination in mouse thyroid tissue monitored by *mTmG* activation using *Nkx2.1* as Cre driver. For control purpose, tamoxifen (tam) was injected (x4) after weaning and animals were sacrified 10 days (d; in D) or 8 weeks (w; in E) thereafter. Excised thyroids were processed for fluorescence microscopy. Cell nuclei were counterstained with DAPI. **A, B**) Non-induced reporter activation after 3 and 6 months is limited to single or small clusters of mG$^+$ cells occasionally present in otherwise mT$^+$ follicles. **C**) Induced reporter activation comprise all cells in the majority of follicles; only few follicles consist of both mG$^+$ and mT$^+$ cells (asterisks). C´ shows high power of a compound follicle (boxed area in C) with heterogeneous mG$^+$ (green arrowheads) and mT$^+$ (red arrowheads) labeling. **D**) Two months after tamoxifen induction, follicles with hetergeneous reporter activation are more numerous due to expansion of both mT$^+$ and mG$^+$ cells (asterisks). Nonetheless, cell proliferation is overall sparse with no signs of tumor formation. Th(R), right thyroid lobe; pt, parathyroid gland. Bars: 500 (C, D), 100 (A, B) µm.
